# Language and nonlanguage factors in foreign language learning: evidence for the learning condition hypothesis

**DOI:** 10.1038/s41539-021-00104-9

**Published:** 2021-09-15

**Authors:** Xin Kang, Stephen Matthews, Virginia Yip, Patrick C. M. Wong

**Affiliations:** 1 Department of Linguistics and Modern Languages, The Chinese University of Hong Kong, Hong Kong SAR, China; 2 Brain and Mind Institute, The Chinese University of Hong Kong, Hong Kong SAR, China; 3 Department of Linguistics, The University of Hong Kong, Hong Kong SAR, China; 4 Childhood Bilingualism Research Centre, The Chinese University of Hong Kong, Hong Kong SAR, China; 5 Department of Otorhinolaryngology, Head and Neck Surgery, The Chinese University of Hong Kong, Hong Kong SAR, China

**Keywords:** Interdisciplinary studies, Human behaviour, Education

## Abstract

The question of why native and foreign languages are learned with a large performance gap has prompted language researchers to hypothesize that they are subserved by fundamentally different mechanisms. However, this hypothesis may not have taken into account that these languages can be learned under different conditions (e.g., naturalistic vs. classroom settings). With a large sample of 636 third language (L3) learners who learned Chinese and English as their first (L1) and second (L2) languages, the present study examined the association of learning success across L1–L3. We argue that learning conditions may reveal how these languages are associated in terms of learning success. Because these languages were learned under a continuum of naturalistic to classroom conditions from L1 to L3, this sample afforded us a unique opportunity to evaluate the hypothesis that similar learning conditions between languages could be an important driving force determining language learning success. After controlling for nonlanguage factors such as musical background and motivational factors and using a convergence of analytics including the general linear models, the structural equation models, and machine learning, we found that the closer two languages were on the continuum of learning conditions, the stronger their association of learning success. Specifically, we found a significant association between L1 and L2 and between L2 and L3, but not between L1 and L3. Our results suggest that learning conditions may have important implications for the learning success of L1–L3.

## Introduction

For decades, linguists, psychologists, neuroscientists, and educators have been puzzled by the observation that young children can learn their native languages with ease, yet adults often struggle to learn even the basics of foreign languages^1–4^. This observation has propelled a large body of research conducted on the hypothesis that native (L1) and nonnative (foreign) (L2) languages are learned, represented, and/or processed in fundamentally different ways that result in this large gap in learning outcomes^5,6^. On the other hand, a smaller but growing set of studies has found similarities in learning outcomes between native and foreign languages, which support the hypothesis that a common set of mental operations may be in place for all language learning^7,8^. By examining the language outcomes of a large sample of learners who have learned three languages consecutively from birth, in early childhood, and in adulthood, the present study aims to evaluate these two sets of hypotheses against a newly proposed hypothesis to offer new insights into native and foreign language learning.

Studies that investigate native and foreign language learning are generally designed to test two sets of hypotheses. The first is the fundamental difference hypothesis (FDH)^5,6^. According to the FDH, native (first) language acquisition relies on a domain-specific core computational system of human language (known as Universal Grammar)^9^, whereas adult foreign language learners either lack access to this innate system or its operation is partial and imperfect, leading to difficult and ultimately unsuccessful outcomes of learning. The central claim of FDH is consistent with the critical/sensitive period hypothesis^10,11^ that native language proficiency could not be achieved beyond a limited age range prior to puberty or young adulthood^12–14^. The age-related decline in language learning ability is said to be part of human brain maturation^15–18^. The second set of hypotheses, including the linguistic coding deficit/differences hypothesis (LCDH)^7,8,19,20^, argue that both native and foreign language learning are tied to the same set of core language functions (e.g., phonological, syntactic, and semantic processing skills), thus learning outcomes for all languages will be interrelated.

The literature on L1 and L2 learning reports two general sets of findings which have been interpreted as supporting either hypothesis. The first is a well-documented set of findings of a large performance gap between L1 and L2, despite years of training for some aspects of morphosyntax and phonology. Even fluent nonnative speakers tend to lag behind native speakers in real-time language processing^21^. These findings are often interpreted as supportive of FDH. The second set of findings concerns not absolute proficiency levels but how performance in the two languages is correlated within individuals^22,23^. For example, Sparks et al.^22^ found that the best predictor of L2 word decoding was individual learners’ L1 word decoding. In addition, auditory processing may explain variability in success in learning linguistic rules by both infants and adults^24^. These results suggest that a common set of core functions may be at play for both native and foreign languages, as suggested by LCDH.

While foundational in contributing to our current understanding of native and foreign language learning, these two sets of studies may not by themselves confirm either of the hypotheses. The first set of studies that was used to support FDH did not examine whether the proficiency of L1 and L2 was correlated but focused on absolute performance levels or the morphosyntax of the languages. The second set of studies was often restricted to the learning of typologically similar languages (Indo-European languages), and the correlation between L1 and L2 was usually found on metalinguistic tasks (e.g., decoding, spelling) with a heavy emphasis on task (procedural) rather than linguistic abilities. The L1–L2 correlation might disappear if the two languages were further apart in typological distance or if metalinguistic abilities were deemphasized.

In the present study, we attempt to address these limitations by examining the association between native and foreign languages and by using more comprehensive measures of language proficiency. We examined the learning of more than two languages differing in typological distance for a more rigorous investigation. Whether a core set of functions subserve the learning of native and foreign languages should be observed in the learning of all languages, not a pair of languages, not only when the languages are typologically close, and not only when a specific task is administered.

Although highly influential, the body of literature evaluating FDH and LCDH may have not considered a crucial aspect of language learning that native and foreign languages can be learned under vastly different conditions. As one of the alternative accounts to FDH and LCDH, we propose the learning condition hypothesis (LCH), in which we postulate that a primary factor determining proficiency levels of languages is the condition under which these languages are learned. Prior research studies have reported two different types of factors that affect language learning success: learner-internal and learner-external factors^25^. Learner-internal factors are about the learners themselves, such as their age^11–17^, nonverbal IQ^26^, and working memory^27^, while external factors refer to stimuli that exist outside the learners such as the environment^28^ and the teacher^29^. Our hypothesis concerns one type of external factors, namely learning conditions. For example, a native language tends to be acquired or learned in a more naturalistic setting with input from caregivers and peers, while foreign language learning usually occurs with explicit instruction and practice in the formal academic context of a classroom. We hypothesize that success in learning one language and the other may be linked, not due to a set of core functions for language learning, but because these languages are learned in similar conditions. Taking the learning of three languages as an example, the three languages could be learned under different conditions and in four orders: (1) L1–L3 being learned naturalistically at home; (2) L1 and L2 being learned naturalistically at home before learning L3 in school; (3) L2 and L3 being learned in school after naturalistic acquisition of the L1 at home; and (4) L1–L3 being learned consecutively on a continuum of naturalistic setting to explicit instruction. Following the LCH, we predict that proficiency in these three languages would be associated differently in the above four situations: (1) proficiency in L1–L3 would all be associated; (2) L1 and L2 would be associated, but not L1/L2 and L3; (3) L2 and L3 would be associated, but not L1 and L2/L3; and (4) L2 would be associated with L1 and L3, but L1 and L3 would not be associated. We suggest that learning conditions exert a stronger effect than factors such as typological distance between these languages^30–33^.

The present study aims to evaluate the three hypotheses: FDH, LCDH, and our newly proposed LCH. We capitalized on our unique ability to access a large population of L3 learners in Hong Kong who learned L1–L3 under different learning conditions. In total, we enrolled 636 participants who were undergraduate students of Chinese descent learning one of three languages as their L3: French (*n* = 187), German (*n* = 176), or Spanish (*n* = 273), at the time of participation. Power calculation was based on the requirements of finding associations between L1 and L2, L2 and L3, and/or L1 and L3 to test our hypotheses. We used the first 25 participants of each L3 to estimate the sample size. We obtained a Pearson’s correlation value between L1 and L2, *r* = 0.25 and for a family-wise alpha of 0.05 (Bonferroni-corrected *p* value of 0.017 for three tests performed to evaluate the relationships between L1 and L2, L2 and L3, and L1 and L3), a minimum of 163 participants in total were required. For each language, we had data available from at least 167 participants for the key measures of L1–L3 proficiency. Our study is therefore sufficiently powered.

On a continuum of naturalistic on one end and instructed on the other, learning conditions of our participants’ L1 and L3 were respectively on the opposite ends, while L2 was in the middle. All participants started to learn Chinese as L1 without the influence of motivational factors from an early age^18^ or even before birth^34^, while they learned English as L2 in the formal education system from ~3 years of age for 15 years through the end of senior secondary education. In Hong Kong, Chinese and English are both official languages. According to the Census and Statistics Department of the Hong Kong SAR^35^, over 90% of individuals aged from 6 to 24 years attending full-time schools could read and write both Chinese and English. However, compared to Chinese, English is by no means to be regarded as another native language for the vast majority of families^36^. Cantonese Chinese remains to be the most commonly spoken language among the majority of the local population and is the main medium of instruction in the formal education system^37,38^, while English input is abundantly available in daily life and can be one source of implicit language input in addition to explicit input from the classroom. Thus, learning conditions of Chinese and English shared differences and similarities in Hong Kong, with Chinese being the L1 acquired naturally but English being the L2 learned mostly in the classroom. Similar to L2, L3 was learned in the classroom and taught by teachers who were native speakers of L3 or nonnative speakers with near-native proficiency. Although both English and L3 were used as the medium of instruction in language classes at the elementary level, all textbooks and handouts were written in L3 only.

**Fig. 1:**
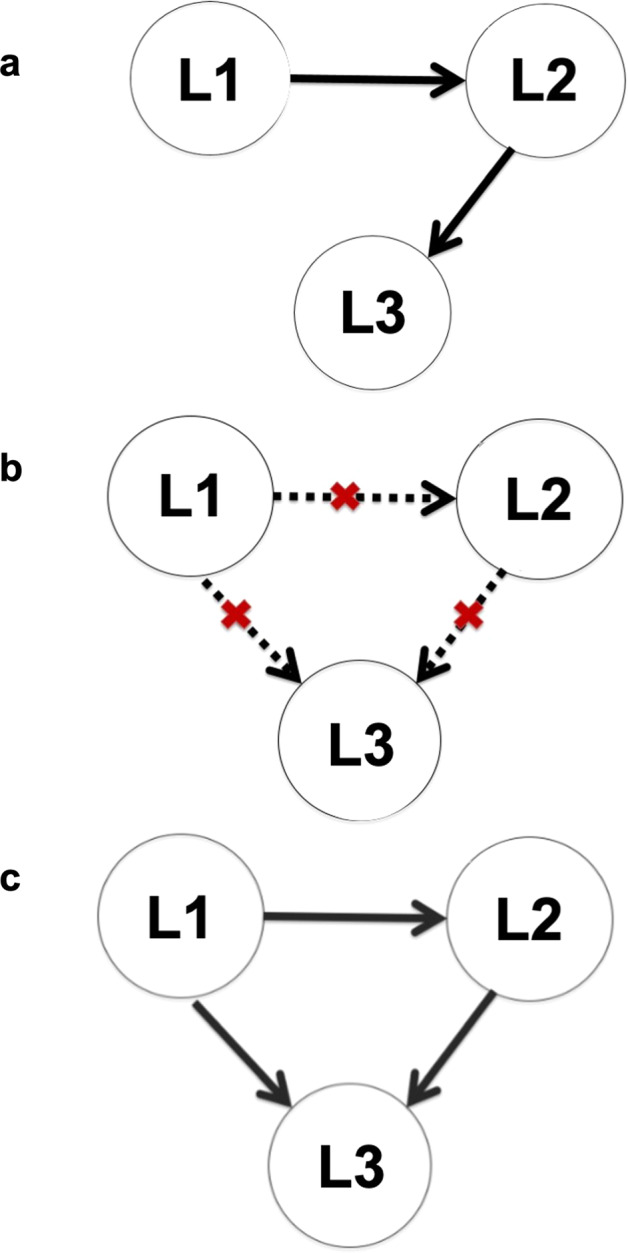
Conceptual metamodels of the relationships between L1, L2, and L3 proficiency. **a** The LCH model postulates that L1 and L2 proficiency and L2 and L3 proficiency are related. **b** The FDH model predicts that L1 proficiency is related neither to L2 nor L3 proficiency. **c** The LCDH model argues that the proficiency levels of all languages are related.

In this study, comprehensive proficiency level for L1 and L2 of participants was assessed by the grades on their college entrance examination, the Hong Kong Diploma of Secondary Education (HKDSE)^39^. L3 proficiency was measured by a combination of measures that included classroom performance and laboratory-based assessment including narrative production, lexical access, and pronunciation judgment. Our study of languages of different typological characteristics also provided an opportunity to examine whether learning conditions as a factor exerted a stronger effect than typological similarity of the languages being learned, as invoked by some theories of L3 learning^32,33^. One objective method of defining typological similarity is by ancestral relationship. Accordingly, English and German (Germanic languages) should be regarded as very close relations and so should French and Spanish (members of the Romance languages group). Germanic languages as a group should be regarded as being closer to Romance languages (the two groups being Indo-European) than they are to a Sinitic language (Chinese). As the contribution of nonlanguage factors may also account for substantial variance in learning, including nonverbal IQ^26^, socioeconomic status (SES)^40–42^, musical background^43,44^, age^11–17^, gender^45^, anxiety^46^, and motivational factors of foreign language learning^47^, we obtained these measures and entered them into our statistical analyses (Table 1). According to FDH, proficiency of L2 or L3 of our participants would not be likely to be related to L1, because L1 is fundamentally different from other languages. According to LCDH, proficiency of all of our participants’ languages should be correlated, because learning of all languages requires the same set of core functions. Importantly, we predict that according to LCH, L3 of our participants should be correlated with their L2, but not their L1, because of the stark difference in learning conditions between L1 and L3, but L2 should be correlated with their L1 due to implicit learning of L1 and L2 in daily life. Figure 1 provides a graphic representation of what each hypothesis predicts.

**Table 1:** Descriptive statistics of participants’ profiles and their L1–L3 proficiency.

Variables	French learners			German learners		Spanish learners			
	Mean (SD)	Range	Ns	Mean (SD)	Range	Ns	Mean (SD)	Range	Ns
Gender (F/M)	141/46		187	120/56		176	210/63		273
Musical training (Y/N)	158/27		185	146/29		175	213/59		272
L3 class level (low/high)	104/83		187	82/94		176	144/129		273
Nonverbal IQ	109 (10)	87–130	185	108 (11)	87–127	175	107 (10)	85–130	269
Family SES	39 (14)	13–66	173	38 (15)	13–65	166	36 (14)	5–66	257
L3 age (years)	19.500 (1.177)	18–25	187	19.830 (1.240)	18–23	176	19.770 (1.177)	18–23	273
HKDSE age (years)	17.460 (0.770)	17–24	148	17.370 (0.543)	17–20	133	17.500 (0.661)	17–20	215
L1 (Chinese)	4.995 (1.011)	3–7	187	4.920 (1.183)	3–7	176	4.916 (1.029)	3–7	273
L2 (English)	5.323 (0.960)	3–7	187	5.148 (0.932)	3–7	176	5.113 (1.026)	3–7	273
L3 Global scores	0.004 (0.985)	−2.210 to 4.734	174	0.014 (1.030)	−2.541 to 3.389	167	0.013 (0.992)	−2.857 to 3.159	244
L3 external motivation	−0.003 (1.046)	−2.240 to 2.572	185	−0.003 (0.982)	−2.051 to 2.648	175	0.006 (0.986)	−2.675 to 2.444	271
L3 internal motivation	−0.027 (0.931)	−3.200 to 2.412	185	0.011 (1.104)	−3.176 to 2.869	175	0.006 (0.975)	−2.849 to 3.067	271
L3 attitude	0.007 (1.009)	−4.800 to 2.316	183	0.001 (1.018)	−2.863 to 2.396	176	0.001 (0.989)	−2.614 to 2.465	269
L3 anxiety	0.075 (1.002)	−2.997 to 2.929	183	−0.031 (0.968)	−2.430 to 2.429	176	−0.035 (1.011)	−3.104 to 2.452	269

L1 (Chinese) and L2 (English) proficiency was represented by the composite grades of the Chinese and English subjects in the HKDSE exam. Music training (Y) = have received at least 1 year of musical training and music training (N) = have received less than 1 year of musical training or have not received any musical training at all. L3 class level (low) = class levels 1 and 2 and L3 class level (high) = class level 3 and above.

*F* female, *M* male, *Ns* Number of participants.

## Results

### Statistical analysis

In order to provide converging evidence for one or more of the three competing hypotheses (Fig. 1), we subjected our data to three types of analyses: general linear models (GLM), structural equation models (SEM) and machine learning (support vector regression, SVR). L1–L3 proficiency measures were obtained from each of the participants. L1 and L2 measures were obtained from the participants’ HKDSE composite scores for Chinese and English subjects^48^. Because our participants learned different L3s and because of a lack of a single standardized measure for these languages, we measured L3 proficiency using a number of classroom and laboratory measures and used statistical data reduction methods to arrive at an L3 Global score for each participant. Regardless of the type of statistical analysis, our primary goal was to demonstrate the degree of association in proficiency between pairs of languages.

### Bivariate correlations

As an initial analysis, we calculated Spearman’s pairwise correlation coefficients (cc) for pairs of L1–L3 (Supplementary Fig. 2; Supplementary Table 3). Statistical significance was indicated by the false discovery rate (FDR) corrected *p* values. L1 (Chinese) HKDSE grades were significantly correlated with L2 (English) HKDSE grades (*r* = 0.26*, p* < 0.001). L2 (English) HKDSE grades were significantly correlated with L3 Global scores (*r* = 0.28*, p* < 0.001). Importantly, L1 (Chinese) HKDSE grades were not significantly correlated with L3 Global scores (*r* = 0.05*, p* = 0.263). These bivariate correlational results provide an initial set of evidence for LCH. Although L1 and L2 were both measured by HKDSE and were significantly correlated, L2 and L3 were significantly correlated despite the differences in measurements.

#### Multiple linear regression models

The bivariate correlational results reported above did not take into account the contributions of other factors that may influence language learning. As discussed in the Introduction, factors such as musical experience could explain variance in language learning^43,44^. We therefore employed multiple linear regression models to explore the relationships among the three languages, with the other nonlanguage factors accounted for. We constructed two separate models. In the first model (Table 2), L2 (English) HKDSE grades were treated as the dependent variable, and L1 (Chinese) HKDSE grades, gender, musical training, family SES, nonverbal IQ, and age were treated as independent variables. We found musical training (*β* = 0.31, *p* = 0.015), family SES (*β* = 0.02, *p* < 0.001), and age (*β* = −0.16, *p* = 0.015) to significantly predict L2 (English) HKDSE grades. Importantly, L1 (Chinese) HKDSE grades also significantly predicted L2 (English) HKDSE grades and exerted the strongest effect of any of the significant predictors (Δ*R*^2^ = 0.06, *p* < 0.001). In the second model (Table 3), L3 Global scores were treated as the dependent variable. In addition to the aforementioned nonlanguage predictors, affective and motivational factors as measured by the modern language (ML) learner questionnaire^49^ were also entered as independent predictor variables, as they have been found to contribute to the learning of a new language (in this study, L1 and L2 were not new languages being learned and we did not know the learners’ motivation of learning L2 since this began in early childhood). Attitude (*β* = 0.13, *p* = 0.043) and age (*β* = −0.08, *p* = 0.044) significantly predicted L3 proficiency. Importantly, L2 proficiency (Δ*R*^2^ = 0.06, *p* < 0.001) was the most significant predictor of L3 proficiency, while L1 proficiency (*β* = −0.02, *p* = 0.580) was not a significant contributor. Taken together, the GLM results indicate an association between L1 and L2 as well as between L2 and L3 after the relevant nonlanguage factors were controlled for. Importantly, we again failed to find an association between L1 and L3.

**Table 2:** Summary of the multiple linear regression model with L2 (English) proficiency as the dependent variable.

Predictors	Estimates	95% CI	*p*	Corrected *p*	Partial eta squared
L1 (Chinese)	0.24	0.16–0.32	<0.001^a,b^	<0.001^a,b^	0.074
Music training	0.31	0.08–0.53	0.008^a,b^	0.015^a,b^	0.015
Family SES	0.02	0.01–0.02	<0.001^a,b^	<0.001^a,b^	0.057
HKDSE age	−0.16	−0.29 to −0.04	0.010^a,b^	0.015^a,b^	0.015
Gender	−0.14	−0.33 to 0.05	0.143	0.163	0.005
Nonverbal IQ	−0.00	−0.01 to 0.01	0.920	0.952	0.000

Variables listed in the first column are independent variables. L1 (Chinese) and L2 (English) proficiency was represented by the composite grades of the Chinese and English subjects in the HKDSE exam. Gender and Music training are dummy coded with 0 = female and 1 = male (Gender) and 0 = have received at least 1 year of musical training and 0 = have received less than 1 year of musical training or have not received any musical training at all (Music training). *R*^2^ = 0.165 (adjusted *R*^2^ = 0.154); *p* of this model < 0.001.

^a^*p* < 0.05 (uncorrected).

^b^Significant associations after FDR corrections for multiple comparisons.

**Table 3:** Summary of the multiple linear regression model with L3 Global scores as the dependent variable.

Predictors	Estimates	95% CI	*p*	Corrected *p*	Partial eta squared
L1 (Chinese)	−0.02	−0.10 to 0.05	0.528	0.580	0.001
L2 (English)	0.28	0.19–0.37	<0.001^a,b^	<0.001^a,b^	0.066
L3 age	−0.08	−0.15 to −0.02	0.012^a,b^	0.044^a,b^	0.012
L3 attitude	0.13	0.03 to 0.24	0.012^a,b^	0.043^a,b^	0.012
L3 internal motivation	0.12	0.02–0.23	0.019^a^	0.052	0.010
L3 anxiety	−0.08	−0.16 to −0.00	0.050^a^	0.109	0.007
L3 external motivation	−0.05	−0.13 to 0.03	0.199	0.313	0.003
Gender	0.11	−0.07 to 0.29	0.247	0.340	0.003
Music training	−0.14	−0.34 to 0.07	0.199	0.355	0.003
Family SES	0.00	−0.00 to 0.01	0.720	0.720	0.001
Nonverbal IQ	0.00	−0.01 to 0.01	0.512	0.580	0.001

Variables listed in the first column are independent variables that have a significant impact on the dependent variable. L1 (Chinese) and L2 (English) proficiency was represented by the composite grades of the Chinese and English subjects in the HKDSE exam. Gender and Music training are dummy coded with 0 = female and 1 = male (Gender) and 1 = have received at least 1 year of musical training and 0 = have received less than 1 year of musical training or have not received any musical training at all (Music training). *R*^2^ = 0.166 (adjusted *R*^2^ = 0.148); *p* of this model < 0.001.

^a^*p* < 0.05 (uncorrected).

^b^Significant associations after FDR corrections for multiple comparisons.

### Structural equation models

#### All participants

**Fig. 2:**
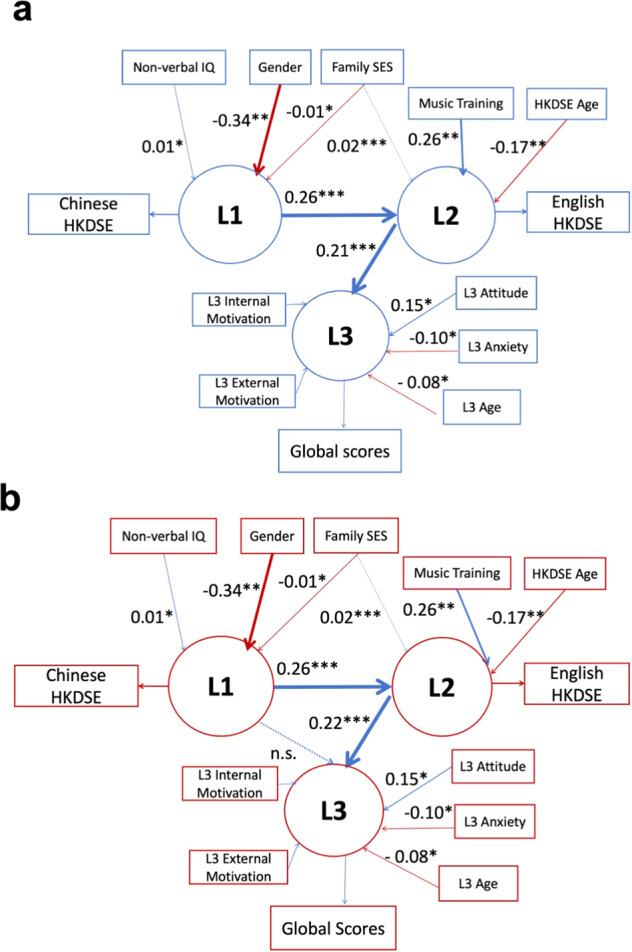
Structural equation models of the relationship among L1–L3 proficiencies along with nonlanguage factors. Parameter estimates are unstandardized, and the paths are scaled to reflect effect size. Red arrows represent negative paths, while blue arrows are positive paths. L1–L3 are latent variables of language proficiency with Chinese HKDSE grades, English HKDSE grades, and L3 Global scores as their indicators, respectively. Only significant relationships are presented and denoted with asterisks: *p < 0.05, **p < 0.01, ***p < 0.001. **a** The LCH model. **b** The LCDH and FDH models are tested simultaneously because they both concern connections (or lack therefore) of L1–L2, L2–L3, and L1–L3. The path between L1 and L3 as indicated by the dashed arrows was not significant.

To evaluate the potential statistical causal links among the three languages while accounting for the contribution of nonlanguage factors, and to directly test the three hypotheses, two latent variable structural equation models were tested. The two models had the same structures except for the paths connecting the three languages. In the first model, paths were drawn from L1 to L2, and from L2 to L3, which enabled us to test LCH (Fig. 1a). In the second model, paths were imposed from L1 to L2, L2 to L3, and L1 to L3. This second model allowed us to simultaneously evaluate FDH (Fig. 1b) and LCDH (Fig. 1c). FDH would predict no statistical effects among any of the paths, while LCDH would predict effects of all three paths. Both LCH and LCDH models provided a statistically acceptable fit. For the first model, the root mean square error of approximation (RMSEA) was 0.025 [CI: 0.000–0.054], the standardized root mean square residual (SRMR) = 0.021, the comparative fit index (CFI) = 0.971, the Tucker–Lewis index (TLI) = 0.946, and the Yuan–Bentler scaling correction factor = 1.019 (Fig. 2a). For the second model, the RMSEA = 0.028 [CI: 0.000–0.058], the SRMR = 0.021, the CFI = 0.967, the TLI = 0.932, and the Yuan–Bentler scaling correction factor = 1.037 (Fig. 2b). While both models were statistically significant, the crucial path between L1 and L3 of the second model was not statistically significant (*b* = −0.021 [CI: −0.101 to 0.059]). Importantly, the fit of the second model showed no significant improvement over the first model (Δ*χ*^2^ = 0.269, *p* = 0.604). Thus, the results suggest that we fail to reject the null hypothesis that the two models were significantly different and thus parsimony would favor the first model that has fewer estimated parameters. These results, demonstrating associations between L1 and L2 and between L2 and L3, but not L1 and L3, support the LCH. Detailed statistics for each path for each model can be found in Table 4.

**Table 4:** Path coefficients of structural equation models (SEMs) for LCH and LCDH.

	LCH			LCDH		
Paths	Estimates	*p*	95% CI	Estimates	*p*	95% CI
L3 latent variable						
L1 latent variable	Not applicable	−0.021	0.604	−0.101 to 0.059		
L2 latent variable	0.210	<0.001	0.113–0.308	0.217	<0.001	0.117–0.316
Age	−0.078	0.027	−0.147 to −0.009	−0.079	0.026	−0.148 to −0.010
Music training	−0.168	0.123	−0.382 to 0.046	−0.170	0.115	−0.382 to 0.042
L3 attitude	0.147	0.015	0.029–0.264	0.149	0.014	0.031–0.267
L3 internal motivation	0.103	0.086	−0.014 to 0.221	0.102	0.090	−0.016 to 0.220
L3 anxiety	−0.097	0.037	−0.189 to −0.006	−0.097	0.036	−0.188 to −0.006
L2 latent variable						
L1 latent variable	0.257	<0.001	0.172–0.324	0.257	<0.001	0.182–0.333
Family SES	0.017	**<**0.001	0.010–0.022	0.017	**<**0.001	0.010–0.022
Music training	0.262	0.039	0.085–0.583	0.262	0.039	0.085–0.583
Gender	−0.165	0.071	−0.340 to 0.011	−0.165	0.071	−0.340 to 0.011
HKDSE age	−0.168	0.007	−0.250 to −0.011	−0.168	0.007	−0.250 to −0.011
L1 latent variable						
Nonverbal IQ	0.011	0.026	0.002–0.021	0.011	0.026	0.002–0.021
Family SES	−0.009	0.010	−0.015 to −0.002	−0.009	0.010	−0.015 to −0.002
Gender	−0.344	0.004	−0.572 to −0.124	−0.344	0.004	−0.572 to −0.124

Gender and Music training were coded as dummy variables with 0 = female and 1 = male (Gender) and 1 = have received at least 1 year of musical training and 0 = have received less than 1 year of musical training or have not received any musical training at all (Music training). Estimates are unstandardized beta coefficients between the two variables indicated by the path. 95% confidence intervals (CIs) were obtained using 10,000 bootstrap resampling method. L1–L3 are latent variables of language proficiency with Chinese HKDSE grades, English HKDSE grades, and L3 Global scores as their indicators, respectively.

#### Separate models for low and high proficiency learners

Our results reported above came from models where all participants were included. It is possible that the results may differ between learners of low and high proficiency levels. We categorized participants into two groups based on the academic class levels that they were enrolled in (see Methods for explanation). We fitted a “free” LCH model with all parameters being allowed to differ between groups. We then fitted a “constrained” LCH model with all parameters being fixed to those obtained from analysis of the pooled data across the two groups. We examined whether the “constrained” model was significantly different from the “free” model. Results suggest that the “constrained” model is not significantly different between low vs. high proficiency groups, Δ*χ*^2^ = 11.62, *p* = 0.637 (Supplementary Fig. 4).

#### Separate models for learners of different languages

Although our study was not specifically designed to test theoretical accounts of L3 learning that are centered on typological similarities^32,33^, we conducted further analysis that separated participants into different L3 language groups. We acknowledge that since we did not explicitly measure the psychotypology as perceived by learners^31^, definition of typological proximity of these languages (Chinese, English, French, German, Spanish) can be controversial. Nonetheless, using ancestral relationship as a measure of typological distance, we may expect the effect between English and German to be strongest since both of them are Germanic languages, which would also exhibit less similarity with French and Spanish (Romance languages), and less similarity with Chinese (a Sinitic language). If language typological distance exerts an effect, we may expect the effect from L2 to L3 to be different across pairs of languages, depending on their typological distance from English. We thus first compared the SEMs of German learners with French learners, and those of German learners with Spanish learners, respectively. In addition to comparisons including German learners as a comparison group, we also examined model differences in the Spanish and French group. Our results revealed that no such comparison is significant (German vs. French: Δ*χ*^2^ = 22.30, *p* = 0.073; German vs. Spanish: Δ*χ*^2^ = 16.73, *p* = 0.271; Spanish vs. French: Δ*χ*^2^ = 11.73, *p* = 0.628) (Supplementary Fig. 5). These results suggest that as far as our large sample of L3 learners and their comprehensive proficiency assessment of the three L3 languages are concerned, associations with learning of English are not significantly related to typological distance. These results are supplementary to our main findings, as our study was not designed to examine the question of typology. Nevertheless, we conclude that learning condition exerts a stronger effect than typology.

### Machine-learning prediction via SVR

**Fig. 3:**
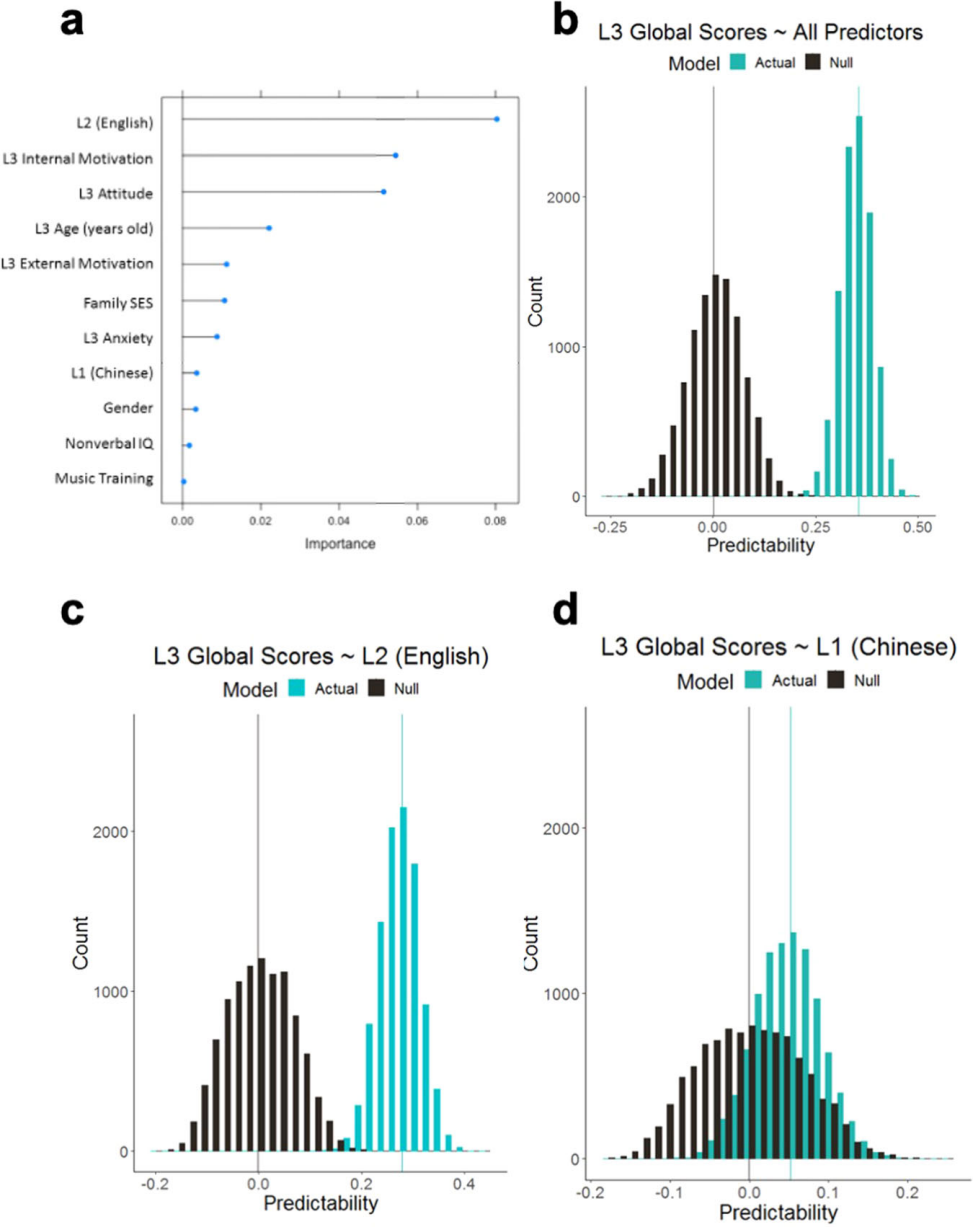
Machine-learning SVR models predict L3 proficiency. The predictability of L3 Global scores was estimated by the correlation coefficients (cc) between the predicted and the observed language proficiency scores based on tenfold cross-validation with 10,000 iterations. **a** The importance ranking of all predictors of L3 proficiency, where the *x*-axis represents the importance value and *y*-axis represents the variables. The importance value was calculated from tenfold cross-validation with 100 iterations. **b** When all predictors are included in the SVR model, the distribution of prediction values was significantly different from the null distribution (*p* < 0.001, Cohen’s *d* = 6.39). **c** With only L2 (English) HKDSE grades as the predictor of L3 Global scores, the distribution of prediction values was also significantly different from the null distribution (*p* < 0.001, Cohen’s *d* = 5.22). **d** With only L1 (Chinese) HKDSE grades as the predictor of L3 Global scores, the distribution of prediction values was significantly different from the null distribution (*p* < 0.001, Cohen’s *d* = 0.96), but the effect size was much smaller than when all predictors or only L2 predictors were included in the models.

Our final analysis involved machine learning using SVR^50,51^. The advantage of this approach is the ability to cross-validate models that are more likely to generalize to future, unseen data, as opposed to traditional GLM approaches that tend to overestimate the true effects^52^. We report Pearson’s cc between predicted and observed outcomes from a tenfold cross-validation procedure with 10,000 iterations. The cc values are used as an indicator of predictability, with higher cc values indicating a more accurate predictive performance. When all predictors were included to predict L3 proficiency in the SVR model (Fig. 3), the predicted cc (mean = 0.355, SD = 0.039) was significantly different from the null distribution (mean = 0.001, SD = 0.068, *p* < 0.001). Importantly, when only L2 (English) HKDSE grades were included as the predictor of L3 Global scores, the distribution of predicted cc (mean = 0.278, SD = 0.039) was also significantly different from the null distribution (mean = −0.001, SD = 0.065, *p* < 0.001). Interestingly, when only L1 (Chinese) HKDSE grades were included as the predictor of L3 Global scores, the distribution of the predicted cc (mean = 0.052, SD = 0.042) again differed significantly from the null distribution (mean = −0.001, SD = 0.065, *p* < 0.001), but the effect size (Cohen’s *d* = 0.96) was at least five times smaller than in the models with all predictors (Cohen’s *d* = 6.39) or only the L2 (English) HKDSE grades (Cohen’s *d* = 5.22) as predictors.

**Fig. 4:**
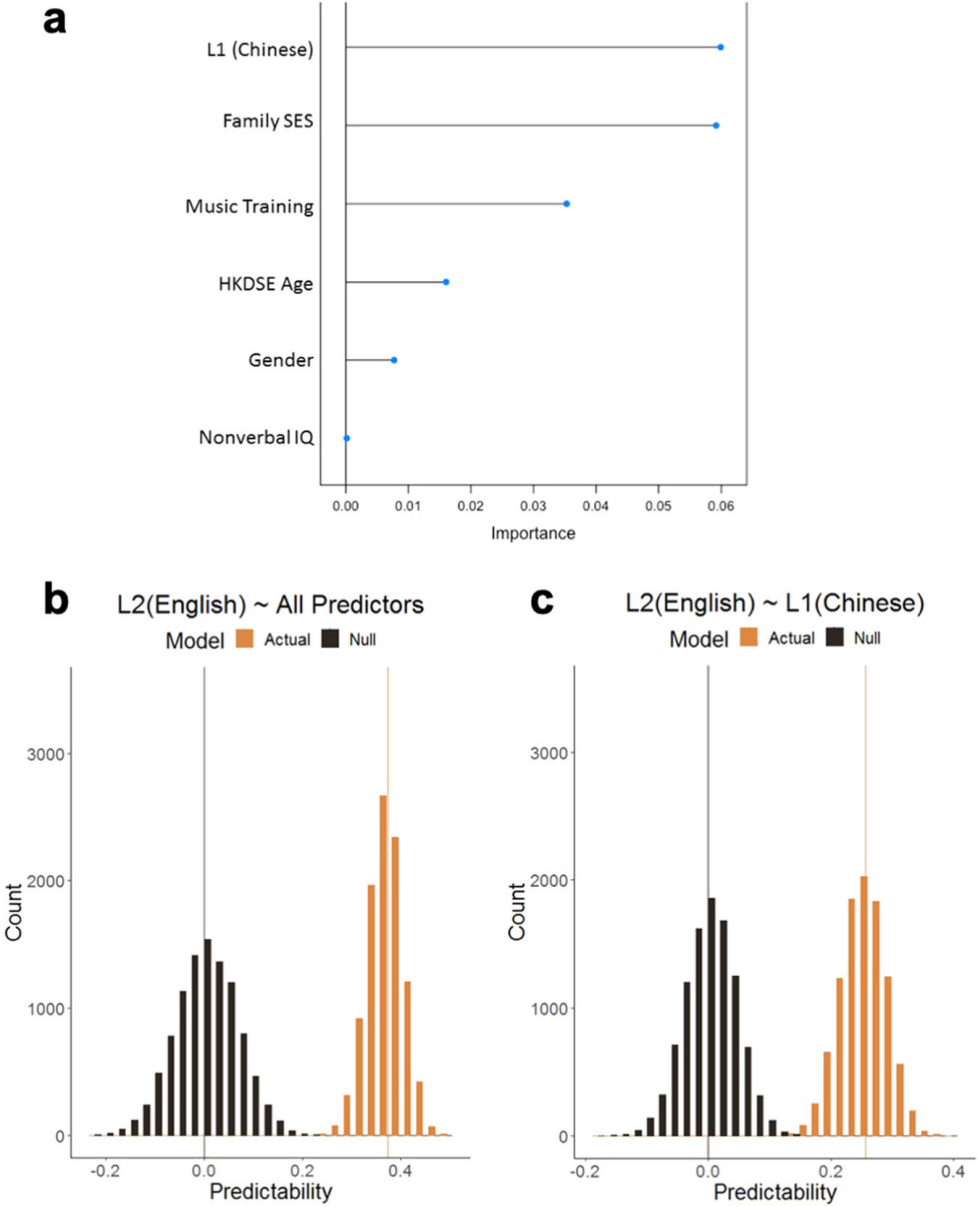
Machine-learning SVR models predict L2 proficiency. The predictability of L2 (English) HKDSE grades was estimated by the correlation coefficients (cc) between the predicted and the observed language proficiency scores based on tenfold cross-validation with 10,000 iterations. **a** The importance ranking of all predictors of L2 proficiency, where the *x*-axis represents the importance value and *y*-axis represents the variables. The importance value was calculated from tenfold cross-validation with 1,000 iterations. **b** When all predictors are included in the SVR model to predict L2 (English) HKDSE grades, the distribution of prediction values was significantly different from the null distribution (*p* < 0.001, Cohen’s *d* = 7.17). **c** With only L1 (Chinese) HKDSE grades as the predictor of English HKDSE grades, the distribution of prediction values was also significantly different from the null distribution (*p* < 0.001, Cohen’s *d* = 6.45).

When all predictors were included in the SVR model to predict L2 (English) HKDSE grades (Fig. 4), the predicted cc (mean = 0.373, SD = 0.036) was significantly different from the null distribution (mean = −0.004, SD = 0.064, *p* < 0.001, Cohen’s *d* = 6.45). When only L1 (Chinese) HKDSE grades were used as the predictor of L2 (English) HKDSE grades, the distribution of predicted cc (mean = 0.257, SD = 0.037) was also significantly different from the null distribution (mean = −0.0004, SD = 0.062, *p* < 0.001, Cohen’s *d* = 7.17).

Taken together, our analysis of data from all 636 participants using GLM, SEM, and SVR approaches supported the LCH hypothesis that predicted significant associations between L1 and L2, as well as between L2 and L3 in this sample of participants.

## Discussion

The present study was designed to examine the relationship of L1–L3 proficiency based on three hypotheses concerning the learning of native and foreign languages. Its focus was on a long-standing academic debate as to whether the learning of all languages depended on a common set of core functions (LCDH) or whether the mechanisms that subserve the learning of native and foreign languages were fundamentally different (FDH). As an alternative theoretical account, we reconceptualized the problem into one that focuses on the learning conditions and postulated that similarities of language learning conditions would result in similarities in learning outcome (LCH), regardless of whether the language to be learned was native or not.

Our access to a large cohort of language learners provided us the opportunity to evaluate the three hypotheses. All participants learned Chinese as L1, English as L2, and either French, German, or Spanish as L3. Learning conditions of L1–L3 ranged along a continuum from naturalistic and implicit (for L1) at one end to instructed and explicit (for L3) at the other, with L2 falling in between. By using four types of analytics (bivariate correlation, regression, SEM, and machine learning), our results converged to demonstrate close relationships between L1 and L2, and between L2 and L3, but not between L1 and L3. Unlike L1 and L2, the participants did not engage in standardized testing for L3. We therefore developed a detailed method for assessing their L3 proficiency by using a number of different classroom and laboratory measures to arrive at an overall L3 Global score using data reduction techniques. It is worth noting that despite differences in how the three languages were measured, a significant association between L2 and L3 was found. The significant association between L1 and L2 was unlikely to be due to measurement similarities.

It is important to highlight that after controlling for nonlanguage factors such as SES^40–42^, musical experience^43,44^, age^11–17^, gender^45^, and motivational factors^47^ that have previously been reported to impact on both native and foreign language learning, we still identified significant associations among the three languages. Our unusually large sample size afforded us the opportunity to look at these factors more closely and control for them statistically. The use of a large sample size and our deployment of multiple types of analytics enhance the generalizability of our findings. Moreover, collecting data from participants who learned a real language in a classroom setting rather than studying an artificial language in the laboratory^53,54^ enhances the ecological validity of our study.

We believe our results cannot simply be explained by the influence of a sensitive/critical period of language acquisition^10–12^. Our learners started L2 acquisition well before any commonly accepted age definition of a critical period for language^11,13,14,16,17^, yet an association between L2 and L3 was found. Our findings are consistent with those of several studies of experience-related neural adaptation in the human brain, namely that the duration and extent of bilingual experiences differentially affects brain structure and function^55–60^. When learning a nonnative language in childhood, learners may tend to approach new information in much the same way as we acquire a native language. For example, Kim et al.^56^ demonstrated that early bilinguals showed overlapping activation for L1 and L2, but segregated activation in late-onset L2 learners. Learning a nonnative language later in life, however, occurs most often in a classroom setting. As with L2 learning, L3 learning may operate under explicit learning conditions and utilize the underlying neural circuitry of nonnative language learning^58^, since the reuse of preexisting mechanisms is consistent with biological and evolutionary principles^61^.

Foreign language learning, namely the acquisition/learning of a language after the first language, is known to be a complex and dynamic experience^62,63^, with individual variabilities in achievement^64,65^. Understanding how nonnative language learning occurs not only enables language teaching to be optimized, with the development of learning and intervention programs that improve learners’ chances of success, but also provides an important context for investigating the interaction of impact factors that may offer a unique and fundamentally important perspective on the biological endowment and neurocognitive adaptations of human beings^55,56^. The present study was not designed to address foreign language learning per se, but as bilingualism/multilingualism is becoming increasingly common, researchers are increasingly interested in the learning of three or more languages. A few theoretical accounts have been proposed to account for L3 learning, such as the typological primacy model (TPM)^32,33^, cumulative enhancement model (CEM)^66^, L2 status factor^67,68^, dynamic model of multilingualism (DMM)^69^, revised hierarchical model (RHM)^70^, linguistic proximity model^71^, and foreign language effect^72^. Nonetheless, these theoretical models focus mostly on morphosyntax (and phonology to a lesser extent) rather than on the overall proficiency level of the learners. They make predictions about whether similarities in structural properties between L2/L3 and L1 or language input would facilitate language learning. The present study was not designed to evaluate any of these three hypotheses concerning L2/L3. In fact, research studies supporting FDH and LCDH have hitherto been usually conducted by focusing on two languages. We believe that by studying the proficiency of L1–L3, our study provides a more rigorous investigation of FDH, LCDH, and LCH. Nevertheless, some of our findings could be interpreted in the context of theories of L2/L3 learning.

The TPM^32,33^ proposes that the language (either L1 or L2) that the learner views as more similar to L3 is the one most likely to facilitate L3 acquisition. The learner determines similarity by first scanning the lexicon, then considering aspects of phonology, and so on. Because English is typologically closer to other Indo-European languages, our finding of a stronger association between L2 and L3 could provide support for TPM as well. However, it is interesting to note that although German is typologically closer to English, we did not find a stronger English–German association in our SEM results than between other L2–L3 pairs, weakening support for the TPM. Furthermore, although Chinese and English are typologically distant, we found a significant association, which we interpreted as a result of learning conditions. The TPM makes no prediction about L1 and L2 association, but it is noteworthy that typological distance alone may not be sufficient to explain all aspects of native and foreign language learning, at least not when a large sample of learners are examined and when overall proficiency level rather specific grammatical structures are studied. We acknowledge that quantifying typological distance is difficult. Reliance on ancestral relationship in our analysis for typological distance could only be a starting point. Nevertheless, it is important to point out that regardless of how typological distance from English is defined (e.g., based on psychotypology)^31^, we found no statistically reliable difference across the L3 languages studied. The effect from English to French was no stronger than the effect between English to Spanish and German.

The CEM^66^ postulates that language learning is cumulative, so that all previous languages (L1 and L2) may have an impact on the learning of a new language (L3). Flynn et al.^66^ examined the production of English restrictive relative clauses by child and adult speakers of Kazakh (L1) and Russian (L2) who learned English as L3. They found subtle differences between adults and children and L1 did not play a more important role in L3 learning. Nonetheless, as the proficiency of L1–L3 was not measured, it was not known from this study whether there was an association between proficiency of these languages.

The L2 status factor^67^ argues that L2 grammar, which is acquired later in life than L1 grammar, exerts a stronger transfer effect than L1 at the initial stages of L3 learning. The latest version of the L2 status factor^68^ specifically argues that similarity in learning contexts and metalinguistic knowledge between L2 and L3, which is most likely subserved by declarative memory, make L2 especially influential. Again, most studies supporting the L2 status factor focused on the grammar rather than the proficiency of learners, but our results of an association between L2 and L3 lend support for this theory to some extent.

The DMM^69^ is another model relevant to the present study. According to DMM, learning of a second language creates a “metalinguistic knowledge and awareness” system that is distinct from that of monolinguals, which facilitates the learning of subsequent languages. The author pointed out that the development of a L3 system is dependent on the dynamic adaptation of existing systems. As our participants learned the three languages consecutively, our results (L1→L2; L2→L3) partly support the claims of DMM, but we did not find any association between L1→L3.

The RHM^70^, a model of bilingual language processing, argues that there are two types of word representations including lexical representations of word forms and conceptual representations of word meanings. Late bilinguals who acquired L2 after early childhood thus showed longer translation latencies from L1 to L2 than from L2 to L1 because of the underlying asymmetry in the strength of the links between lexical and conceptual representations in L1 and L2. In our data, it could be that the links between L2 and L3 were stronger than between L1 and L3, because L2 was also used as a language of instruction. However, as we demonstrated in our analysis, both lower level learners and high level learners showed converging patterns despite the differences in the percentages of L2 used in the teaching of L3.

One limitation of this study is that although we have investigated a relatively large set of nonlanguage variables, these may not fully represent all factors that influence language learning. For example, we have not examined the impact of different types of memory (including the procedural/declarative system^61^, working memory^27^, or language ‘aptitude’^73–75^) on the success of L3 learning. Future studies should attempt to explore a broader range of variables, such as language aptitude, working memory, procedural memory, and declarative memory, to expand our understanding of the interaction between language and nonlanguage factors. Furthermore, as participants in our study learned both their second and third languages in the classroom, instead of (as with their native language) by natural immersion, future studies might profitably examine the cases of immigrants or heritage speakers, who share the context of the native and the nonnative language. This could provide additional evidence for the LCH model. In addition, we acknowledge that age of learning does covary with the continuum of learning conditions, and thus could be a confounding variable. Our learners started learning L1 from birth, but L2 and L3 at around 3 years and 18 years, respectively. Despite the relatively small age difference between L1 and L2 (3 years), the strength of their association of learning success is comparable to the association between L2 and L3 where there is an age gap of around 15 years. Future research will need to systematically address age of learning as a contributing factor, but from the evidence available it does not appear that age is a primary contributor to the results (see also Flege et al.^15^). Another limitation of our study is that it did not account for the potential influence of genetic variation on the learning of native and foreign languages^51,76–78^, which should be addressed in future research.

In sum, the current study adds to the growing body of evidence demonstrating the influence of prior linguistic experience, motivational and affective factors on the learning of a new nonnative language. Importantly, the study provides supportive evidence for the hypothesis that learning conditions of languages may be the principal factor that influences how the proficiency levels of L1/L2/L3 are associated. As shown by our participants from Hong Kong, their L1 proficiency had a positive effect on their L2 but not on their L3, while their L2 had a positive effect on their L3, even after nonlanguage factors are accounted for. Our results provide empirical evidence for our current understanding of language learning and broader issues of human cognition and learning. The results may have implications for studies concerning intervention for communication disorders in children^79,80^ and adults^81^.

## Methods

### Participants

A total of 636 participants between 18 and 25 years of age were enrolled in the present study. They were all native speakers of Cantonese who learned English as a second language from early childhood. All were students of a ML class at the Chinese University of Hong Kong, who were learning either German (*n* = 176), French (*n* = 187), or Spanish (*n* = 273) as L3. All participants had nonverbal IQ within normal limits (≥85), as assessed by the Test of Nonverbal Intelligence, Fourth Edition^82^. Hearing was screened for the frequencies of 500 Hz, 1 kHz, 2 kHz, and 4 kHz at 30 dB HL in a sound booth. Participants supplied basic demographic information, including gender, date of birth, and family SES by completing a questionnaire, and also answered questions on their musical and language background. To calculate musical training experience, we asked participants whether they had received musical training before and listed the style of music they studied (e.g., jazz piano) and years of training undertaken in that particular style. We coded participants’ musical training into two categories: Yes = have received at least 1 year of musical training and No = have received less than 1 year of musical training or have not received any musical training at all. Family SES was assessed following the Hollingshead index^83^, an extensively used measure, by coding parents’ educational levels and occupational prestige. Participant characteristics are reported in Table 1. Not every participant had data from all of these measures. Missing data were randomly presented in the dataset due to incomplete data submission by the participants or coding errors. Written informed consent was obtained from each participant. The research protocol was approved by the Joint Chinese University of Hong Kong–New Territories East Cluster Clinical Research Ethics Committee. Participants were invited to join the study through mass emails and advertisements in their language classes after obtaining permission from the language teachers.

### L1 and L2 proficiency measure

Standardized public examinations are commonly adopted as measures of language proficiency in large-scale research^84^. We used participants’ composite grades of Chinese and English language subject in the HKDSE examination as L1 and L2 proficiency scores. The composite score is calculated based on reading, writing, speaking, and listening skills. HKDSE examination is the public examination for university entrance in Hong Kong, administered by the Hong Kong Examinations and Assessment Authority^48^. Standards referenced reporting with annual calibration exercises is implemented to ensure that scores across years reflect the same levels of performance^39^. Participants’ average grade of 5.32 on the English subject test is roughly equivalent to an overall band score around 7 in the International English Language Testing System. HKDSE examination is typically taken in the final year of secondary school (at around age 17 years).

### L3 proficiency measure

Unlike L1 and L2, our participants did not attend any standardized public examination for L3. This presents a challenge for obtaining an overall measure of L3 proficiency, especially when different languages were learned. To overcome this challenge, we obtained laboratory-based and classroom-based measures from each participant which covered their reading, writing, speaking, and listening abilities for their target language. In the laboratory, participants provided a narrative sample by telling the “Frog, Where Are You?” story^85^. Their production was then transcribed and analyzed using the CLAN program following the CHAT transcription manual^86^ for a number of narrative measures (see Supplementary Table 1). For lexical access, participants named body parts in the target language using the Hawaii Assessment of Language Access battery^87^. For assessment of the native accent of speech production, short excerpts from the narrative production were evaluated by native speakers. We also had access to each participant’s exam score for the language class they took. These exam grades were z-transformed in order to be compared across classes. All of these measures were subject to a data reduction procedure via principal component analysis (PCA), and the final L3 Global score was obtained for each participant.

#### L3 narrative measures

Participants were instructed to tell a story in the target languages based on pictures taken from a children’s wordless story book named “Frog, Where Are You?”^85^. A microphone recorded the storytelling. No time limit was set for this task. Audio recordings were transcribed by native speakers of the target languages using the CLAN program by following the CHAT transcription manual^86^. Two transcribers were employed for each language. The first transcriber transcribed the audio recordings, while the second transcriber checked the transcript and coded learners’ errors at the word and sentence levels. The second transcriber also randomly transcribed 10% of the audio recordings from scratch for a reliability test. On average, a 93% consensus was reached by comparing the first-draft transcripts from the first and the second transcribers after removing punctuations. All transcripts were double-checked by research assistants who spoke the target languages as an additional quality control procedure. Discrepancies were noted and resolved by communicating with transcribers. The transcripts were then automatically coded with morphosyntactic information using the CLAN program^86^. In total, the transcripts contained 70,020 words of French, 65,804 words of German, and 82,904 words of Spanish. The vast majority of these words (93.33%, 96.32%, and 97.18%, respectively) were automatically tagged by the CLAN program.

A PCA was conducted on the 15 indexes of the quality of L3 narrative production (see Supplementary Table 1) with orthogonal rotation (varimax). The Kaiser–Meyer–Olkin (KMO) measure verified the sampling adequacy for the analysis (measure of sampling adequacy (MSA) value = 0.78) with all variables having MSA above 0.50 as the cut-off point. Bartlett’s test of sphericity, *χ*^2^ = 1768, *p* < 0.001, indicated that correlations between items were sufficiently large for PCA. An initial analysis was run to obtain eigenvalues for each component in the data. Three components had eigenvalues above Kaiser’s criterion of 2 and in combination explained 65% of the variance. The scree plot showed inflexions that would justify retaining two components in the final analysis. Factor loadings suggest that component 1 represents length of the narrative, component morphosyntactic complexity, and lexical diversity, while component 2 represents the content, including the mean length of the utterances (Supplementary Fig. 1; Supplementary Table 2)

#### L3 language access

In addition to linguistic knowledge, research in bilingualism has examined the relative strength of the two or more languages by considering language access. Based on psycholinguistic principles, language access can be defined by the relative speed of accessing and naming basic vocabulary and simple phrase structures. Participants were assessed using a picture naming task following the Hawaii Assessment of Language Access battery^87^. Participants were instructed to name 31 photographs of body parts in the target language as quickly as possible. The stimuli were presented in a random order on a computer screen. Participants pressed a button on the response box to present the next stimulus. We recorded what they named and calculated accuracy rates of naming as an indicator of L3 vocabulary. Native speakers of the target languages listened to the audio recordings and judged whether participants gave an accurate name of the picture. We calculated the percentage of accuracy rates for each participant (Supplementary Table 2).

#### L3 pronunciation ratings

Participants were rated for their pronunciation by native speakers of the target languages who had either no exposure, or very limited exposure, to Cantonese. Two 20–30 s excerpts per recording were taken from the beginning and the end of each recording, excluding any initial pauses or false starts. Two counterbalanced lists were created for each language. Each list was further divided into subquestionnaires with 30–40 trials per list. Sixteen native speakers were recruited to rate the recordings on a 9-point scale for native-like qualities via crowd-sourcing programs. The recordings were presented to them in randomized order via Qualtrics.com. The final ratings were averaged across mean ratings of the two excerpts (Supplementary Table 2).

#### L3 classroom exam scores

To measure participants’ classroom performance, the final exam scores of the L3 language were collected at the end of each academic term. A typical exam consisted of speaking, writing, listening, and reading. For each language class, permission was obtained to gather the mean and standard deviation of the final exam for the entire class. We were therefore able to convert the raw exam scores of each study participant into a *z* score that reflected their relative performance within the class that they took (Supplementary Table 2). Because of the various limitations of relying solely on classroom exams to assess student performance^88,89^ and because different languages and proficiency levels were compared, classroom exam performance was only one of the many measures we considered in arriving at the final L3 Global score for each participant.

#### L3 Global score

The procedures described above generated five measures associated with L3 outcome: the first two components from the PCA of the narrative analysis, language access, pronunciation, and classroom exam. To eliminate the variability of language proficiency across participants who enrolled at different class levels of the third languages, we standardized narrative measures, language access, and pronunciation ratings by calculating the *z* scores within each class level of each third language. We then entered these four measures together with classroom exam scores into a PCA with orthogonal rotation (varimax) for further data reduction. The KMO measure verified the sampling adequacy for the analysis (MSA value = 0.60) with all variables having MSA above 0.50 cut-off point. Bartlett’s test of sphericity, *χ*^2^ = 144.99, *p* < 0.001, indicated that correlations between items were sufficiently large for PCA. An initial analysis was run to obtain eigenvalues for each component in the data. We took the loadings of the first component that had an eigenvalue of 1.58 and explained 32% of the variance as the L3 Global scores to mark the overall L3 proficiency of the participants (Supplementary Fig. 1).

Supplementary Fig. 2 and Supplementary Table 3 show Spearman’s rho correlations between L3 Global scores and the five L3 outcome measures inputted into the original PCA analysis. In addition, correlations between L3 measures and L1 and L2 proficiency are also reported.

### ML learner questionnaire

Success in learning a new language is correlated with the learners’ motivation, which can be measured by the ML learner questionnaire^49^. The original questionnaire was designed to cover ten factors, including ideal L2 self, ought-to L2 self, family influence, and attitudes in the first two parts of the questionnaire. Entering all ten factors into our statistical analysis would be inappropriate, and we employed a data reduction method to identify fewer underlying variables. Using PCA with varimax rotation, two components were retained for Part I of the questionnaire. One covered factors related to extrinsic variables such as ought-to L2 self and family influence, which we labeled “external motivation.” The other, which we labeled “internal motivation,” included items on Ideal L2 Self. A separate PCA analysis was conducted for Part II of the questionnaire and two factors, named anxiety and attitude, were retained (Supplementary Fig. 3a, b).

As learners’ motivation is significantly associated with the outcome of learning a new language, motivation was measured in detail by adapting the ML Learners’ motivation questionnaire^49^. The first part of the questionnaire consists of 49 statement-type items measuring the learners’ motivation (e.g., “I have to learn ML because I don’t want to fail the ML course”). Participants were asked to give their ratings on a six-point Likert scale, with the options ranging from “Strongly disagree” to “Strongly agree.” The second part consists of 17 question-type items about learners’ anxiety and attitudes toward the target language class, the native speakers’ community, and the culture (e.g., “Do you always look forward to ML classes?”). Participants were instructed to give their answers on a six-point Likert scale, with options ranging from “Not at all” to “Very much.”

Separate PCAs based on varimax rotation were conducted on Part I and Part II of the learner motivation questionnaire, as illustrated in Supplementary Figs 3 and 4. For Part I (Q1–Q49), the KMO measure verified the sampling adequacy for the analysis (MSA value = 0.93) with all variables having MSA above 0.50 as the cut-off point. Bartlett’s test of sphericity, *χ*^2^ = 1950, *p* < 0.001, indicated that correlations between items were sufficiently large for PCA. An initial analysis was run to obtain eigenvalues for each component in the data. Two components had eigenvalues above Kaiser’s criterion of 4, and in combination explained 35% of the variance. The scree plot showed inflexions that would justify retaining two components in the final analysis. Factor loadings suggest that component 1 represents external motivation, and component 2 internal motivation (Supplementary Fig. 3a).

For Part II (Q50–Q67), we followed the same procedure of data analysis. The KMO measure verified the sampling adequacy for the analysis (MSA value = 0.86) with all variables having MSA above 0.50 as the cut-off point. Bartlett’s test of sphericity, *χ*^2^ = 588, *p* < 0.001, indicated that correlations between items were sufficiently large for PCA. An initial analysis was run to obtain eigenvalues for each component in the data. Three components had eigenvalues above Kaiser’s criterion of 2, and in combination explained 42% of the variance. The scree plot showed inflexions that would justify retaining two components in the final analysis. Factor loadings suggest that component 1 represents attitudes toward the L3 language, culture, and community, while component 2 represents anxiety (Supplementary Fig. 3b).

### Statistical analysis

#### Data reduction

As many measures about L3 proficiency were obtained from each participant, we first employed data reduction procedures using the PCA to reveal major components of the overall language proficiency of each participant (henceforth “L3 Global scores”).

#### Correlations between L1, L2, and L3 proficiency

Spearman’s cc and *p* values were calculated between indicators of language proficiency of L1–L3 using R^90^. Pairwise deletion was adopted to minimize loss through listwise deletion. FDR correction was used to calculate statistical significance.

#### General linear regression models

We fitted separate general linear regression models for L2 and L3 proficiency. To predict English HKDSE grades, we included demographic factors and Chinese HKDSE grades as predictors (Table 2). To predict L3 Global scores, we used not only demographic variables but also motivational and affective factors (internal motivation, external motivation, anxiety, and attitude), along with both Chinese and English HKDSE grades as predictors (Table 3). We used the “p.adjust” function in R, and calculated FDR adjusted *p* values with the Benjamini–Hochberg method.

#### Structural equation models (SEMs)

To further quantify statistical relationships among L1–L3 proficiency, we fitted a series of latent variable structural equation models (SEMs)^91,92^ to allow simultaneous fitting of multiple regression models using the lavaan package, version 0.6-1^93^ in R^90^. We assumed a causal structure of predictor variables and hypothesized two a priori metamodels to evaluate our three hypotheses The LCH model predicts that L1 has effects on L2 but not on L3, while L2 has effects on L3 (Fig. 1a). FDH predicts no relationship across the three languages (Fig. 1b). The LCDH model hypothesizes that L1 has effects on both L2 and L3, while L2 has effects on L3 only (Fig. 1c).

In all models, proficiency scores in L1–L3 were treated as latent variables that were approximated using scores from exams and experiments. Using language proficiency scores as latent variables, we fitted a structural model to reflect the hypotheses about how L1–L3 are related to each other. The measurement model links the latent variable to the observed variables. Exam grades of Chinese (L1) and English (L2) subjects in the HKDSE were used as observed variables to measure L1 and L2 proficiency, respectively. L3 Global scores, calculated using the PCA based on lab and classroom measures, were used as the observed variable of L3 proficiency. Demographic, music, IQ, and motivational factors were also added to the measurement models.

#### SEM model selection and parameter estimation

We fitted two separate latent variable SEMs to test the hypotheses of LCH and LCDH, using full information maximum likelihood to adjust for missing data, and with the robust SEs to account for nonnormality. The SEM of LCH was trimmed to achieve better global fit statistics by removing paths with high standard residuals. We then adopted the same structure of the LCH SEM by including a path between the latent variable L1 and L3 as the model of LCDH. We assessed the goodness of fit for each model and reported the parameters for the most likely model. To evaluate the overall fit of the models, we used the CFI (acceptable fit: 0.95–0.97, good fit: >0.97), SRMR (acceptable fit: 0.05–0.10, good fit: <0.05), and RMSEA (acceptable fit: <0.08, good fit: <0.05), and reported the TLI (good fit: ≥0.80) and Yuan–Bentler scaling factor for each model^94,95^. We did not use the *χ*^2^ of overall fit, because the test is always rejected when the sample size is large.

#### Machine-learning prediction analysis

We conducted prediction analyses using SVR classifier under support vector machine to examine whether proficiency of L2 and L3 can be predicted by significant predictors in the GLMs. SVR was implemented with the e1071 package^50^ in R^90^. We obtained algorithms of SVR models with linear kernel and the penalty parameter C = 1 and epsilon = 0.1 to predict language proficiency. We calculated the mean of the predictions of the SVR models and used this average as our global projection of language proficiency. To evaluate the performance of the SVR, we used a nested tenfold cross-validation procedure via the e1071^50^ and caret^93,96^ packages in R^90^. In this procedure, we first randomly used data from 90% of the participants to build a training SVR model. Parameters from the training model were used to predict the language proficiency of the remaining 10% of participants as test data. For each iteration, we calculated the Pearson’s cc between the predicted language proficiency and the actual language proficiency in the test data. We repeated this process ten times and calculated the averaged cc. We then repeated this process 10,000 times, resulting in a distribution containing 10,000 cc values which represents the SVR model’s performance of predicting language proficiency. A null distribution of predictability was generated by a permutation test based on randomly ordered observed data. We repeated this permutation procedure 10,000 times with the same tenfold cross-validation procedure to generate the distributions for the predictability of the null model. Overall, 5% was set as the critical value for a two-tailed *t* test of the null hypotheses (predictability being the same as random predictions).

### Reporting summary

Further information on research design is available in the Nature Research Reporting Summary linked to this article.

## Supplementary information

Supplementary Information

Reporting Summary

## Data Availability

All data needed to evaluate the conclusions in the paper are present in the paper. The numeric data of this study are available at Open Science Framework (https://osf.io/f5wt8).

## Code availability

The script for statistical analysis is available is available at Open Science Framework (https://osf.io/f5wt8), for purposes of reproducing or extending the analysis.
