# Supplementary material for: Language and nonlanguage factors in foreign language learning: evidence for the learning condition hypothesis
Source: NPJ Sci Learn. 2021 Sep 15;6:28. doi: 10.1038/s41539-021-00104-9 (PMC8443555; doi:10.1038/s41539-021-00104-9)
Supplement: Supplementary file 1 — Supplementary Information [file 41539_2021_104_MOESM1_ESM.pdf]

Supplementary Information

Supplementary figures and tables

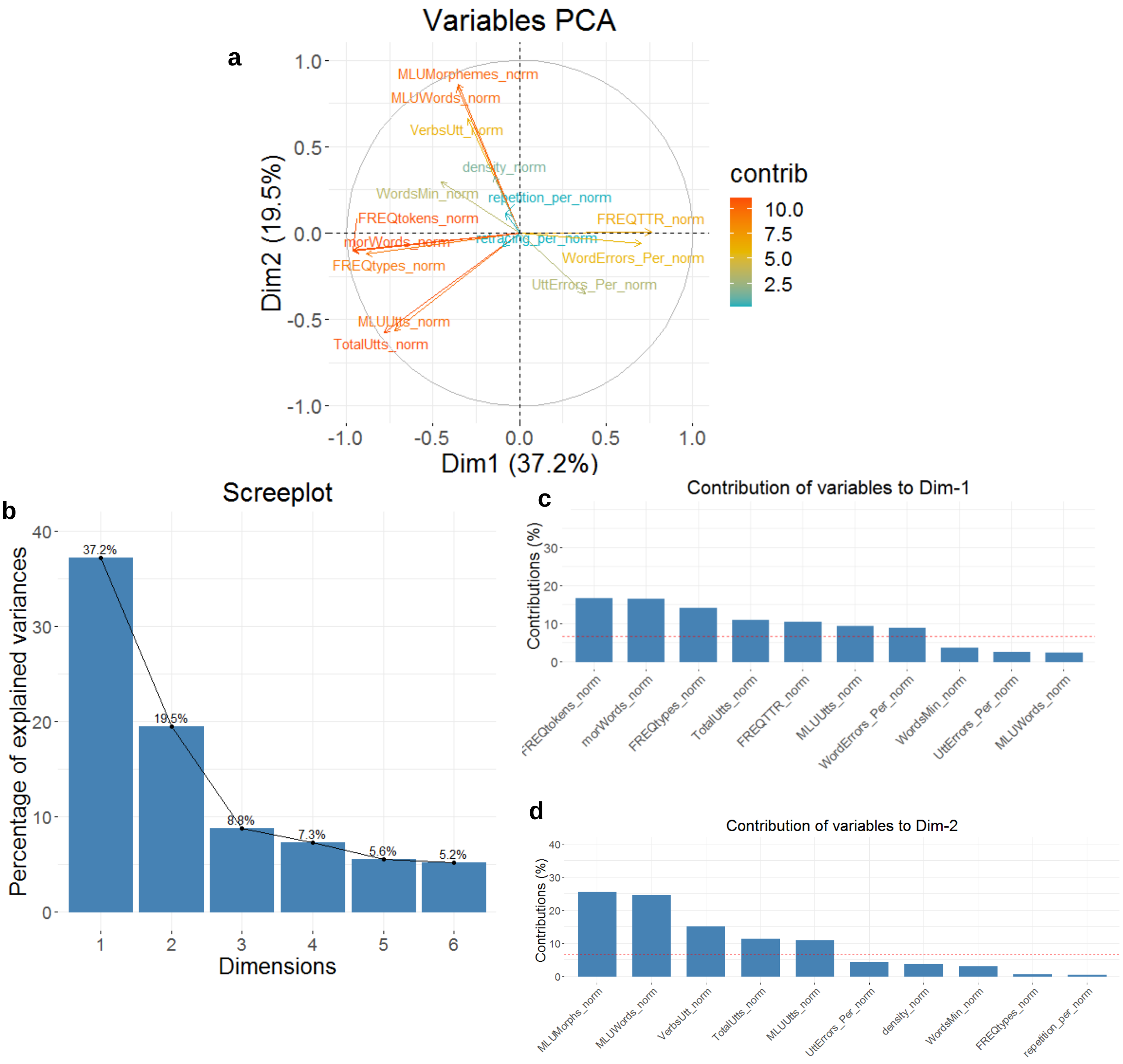

Supplementary Figure 1: PCA results of the L3 narrative measures. a) Two components explained over 56.7% of the total variances. Tb) The scree plot showed inflexions that would justify retaining two components in the final analysis. c) Factor loadings suggest that component 1 represents length of the narrative, component morphosyntactic complexity and lexical diversity. d) component 2 represents the content, including the mean length of the utterances

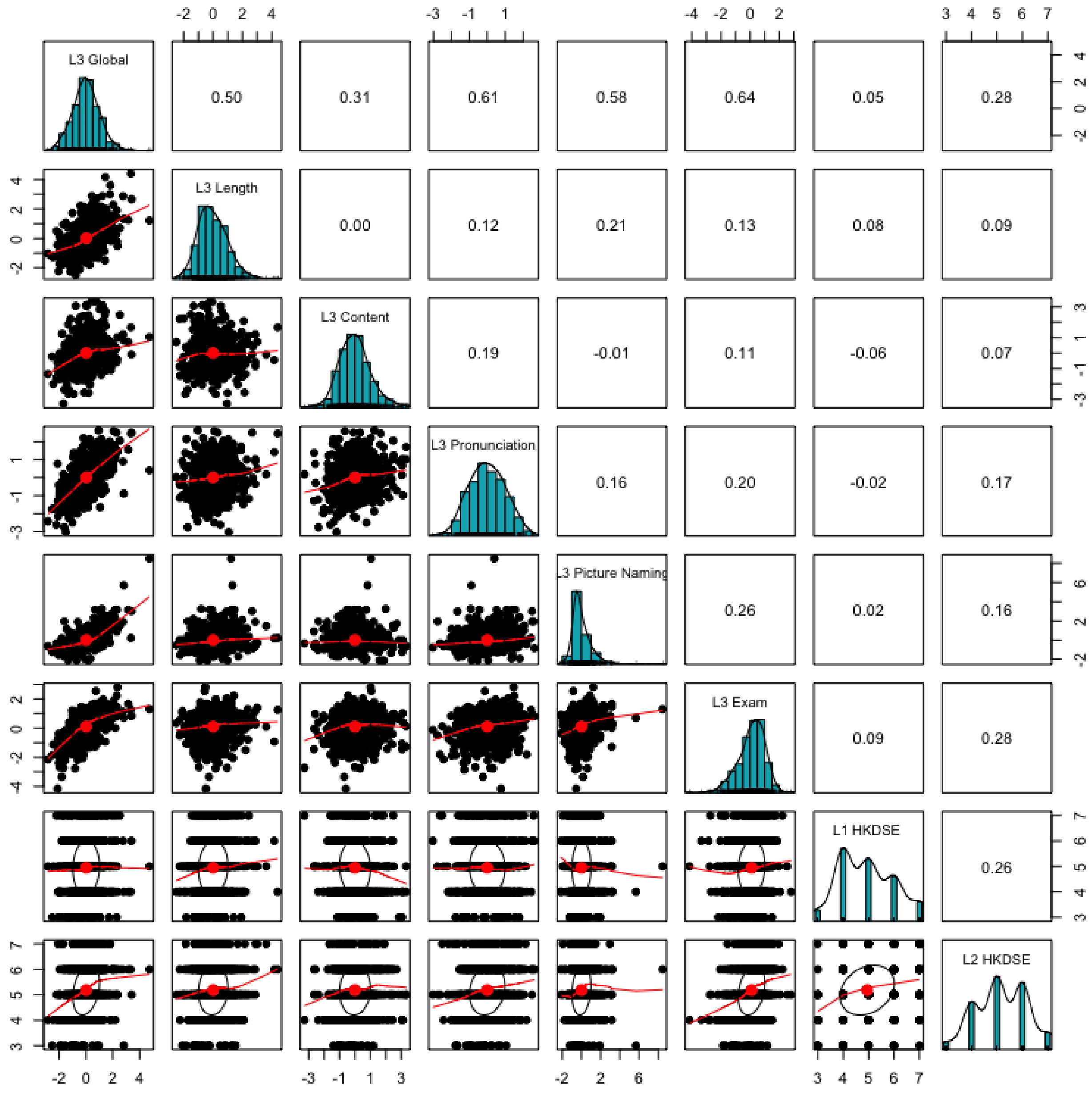

Supplementary Figure 2: The correlation matrix of L1, L2, and L3 outcome measures. The corresponding names of these measures and their distributions are shown in the boxes with histograms. The L1 and English HKDSE grades were treated as ordinal variables. The values in the boxes on the right side of the panel indicate Spearman's rho correlation coefficients. The scatterplots in the boxes on the left side of the panel illustrate the bivariate correlations.

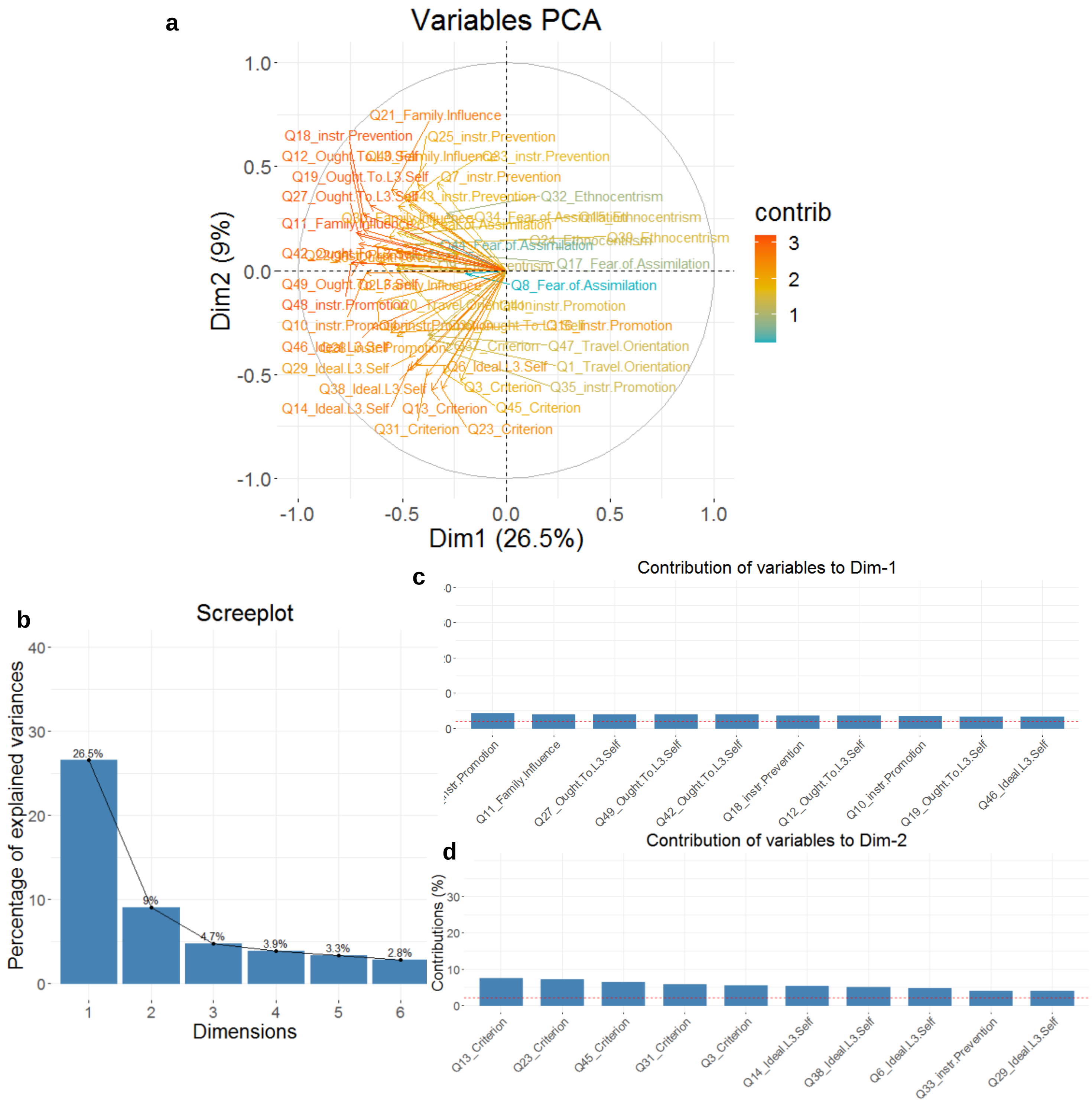

Supplementary Figure 3a: PCA results of the first section in the learners' motivation questionnaire. a) Two components explained over 35.5% of the total variances. b) The scree plot showed inflexions that would justify retaining two components in the final analysis. c) Component 1 represents external motivation. d) Component 2 represents internal motivation.

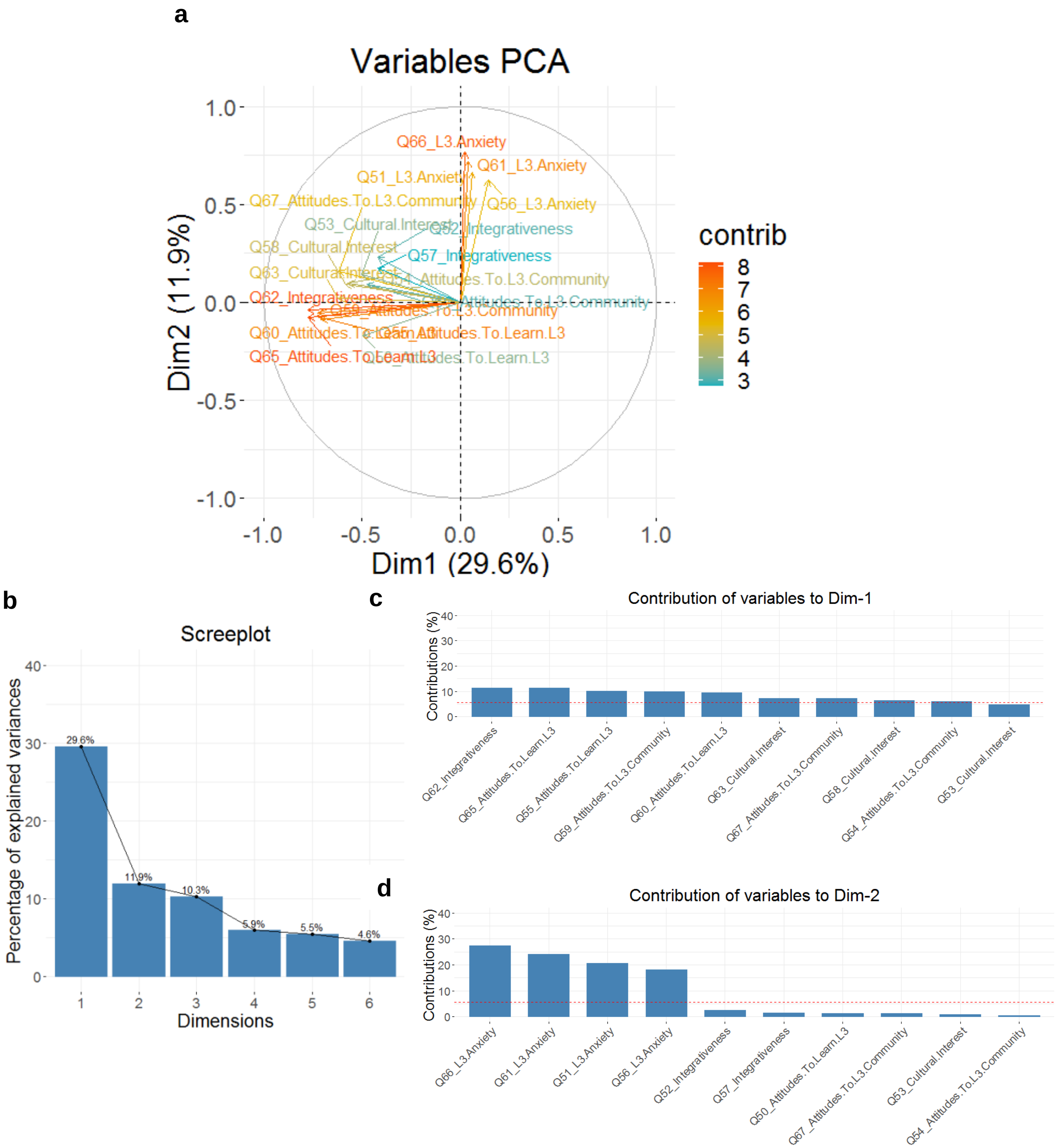

Supplementary Figure 3b: PCA results of the second section in the learners' motivation questionnaire. a) Two components explained over 40.5% of the total variances. b) The scree plot showed inflexions that would justify retaining two components in the final analysis. c) Component 1 represents attitudes towards the L3 language, culture and community. d) Component 2 represents anxiety.

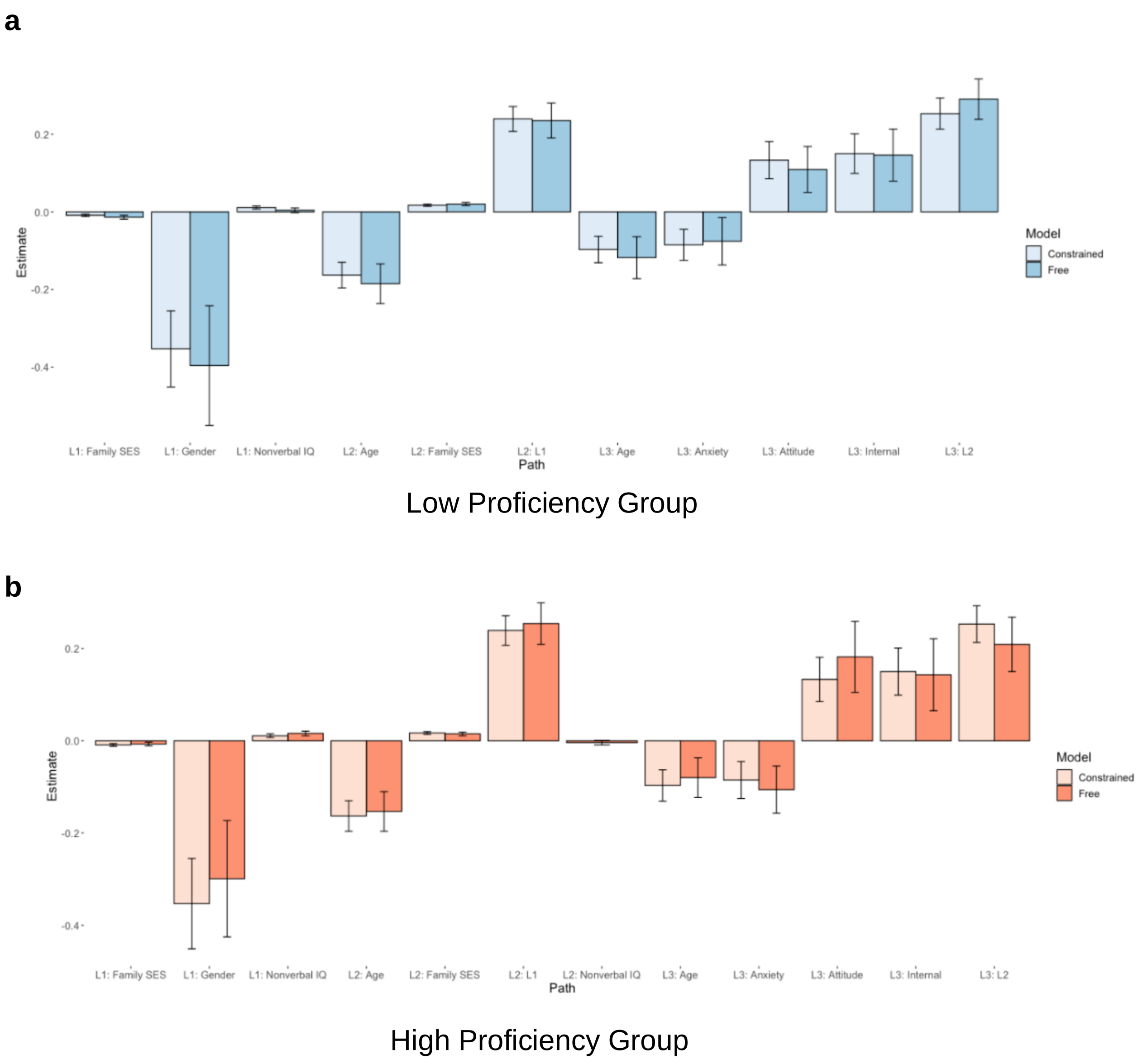

Supplementary Figure 4: Contrasts of path coefficients of the “free” and “constrained” LCH models with proficiency levels (low vs. high) as the group comparison factor. a) Path coefficients of the low proficiency group; b) Path coefficients of the high proficiency group. In the “free” model, all parameters are allowed to differ between low vs. high proficiency groups. In the “constrained” model, each path, regardless of its group, is calculated from the pooled data across low vs. high proficiency groups. Since the two models did not significantly differ and the “constrained” model fitted the data well, we assume that there is no variation in the path coefficients by low vs. high proficiency (refer to the Main Text for further description of the data). Error bars indicate standard errors.

a

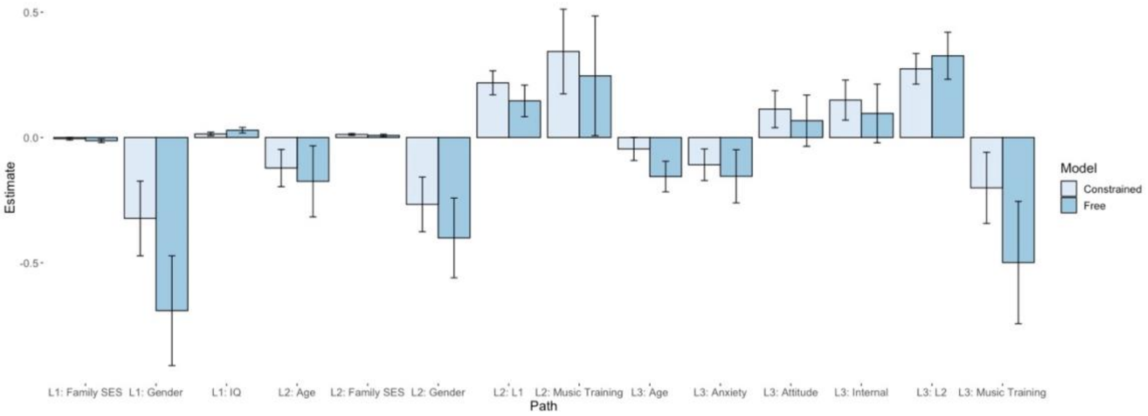

German Learners

b

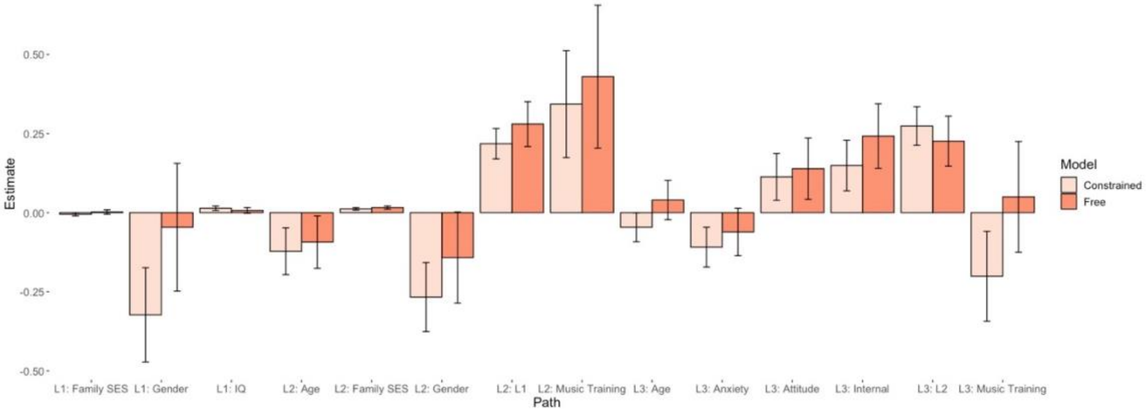

French Learners

Supplementary Figure 5a: Contrasts of free" and "constrained" LCH models with L3 languages as the group comparison factor. a) German learners from the German vs. French groups. b) French learners from German vs. French groups. Each comparison included a "free" model, in which all parameters are allowed to differ between the groups, and a "constrained" model, in which each path, regardless of its group, is calculated from the pooled data across groups. Since the two models did not significantly differ and the "constrained" model fitted the data well, we assume that there is no variation in the path coefficients depending on the L3 languages (refer to the Main Text for further description of the data). Error bars indicate standard errors.

a

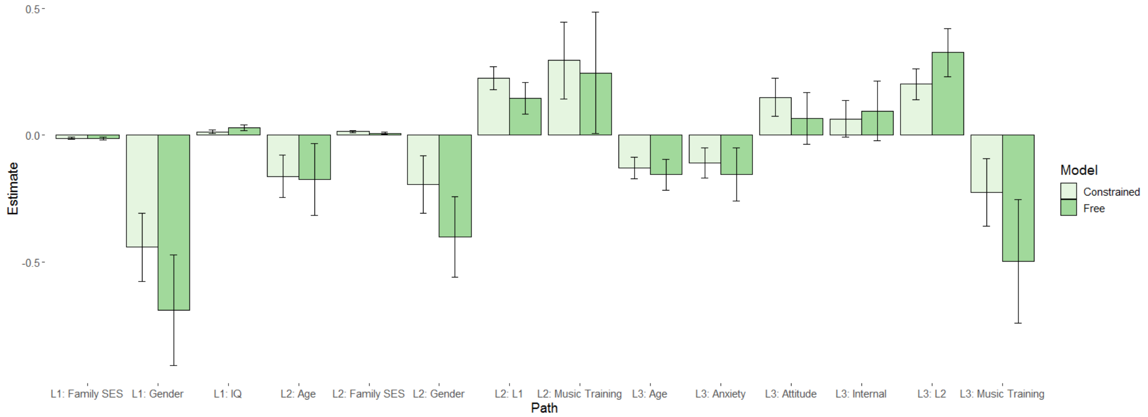

German Learners

b

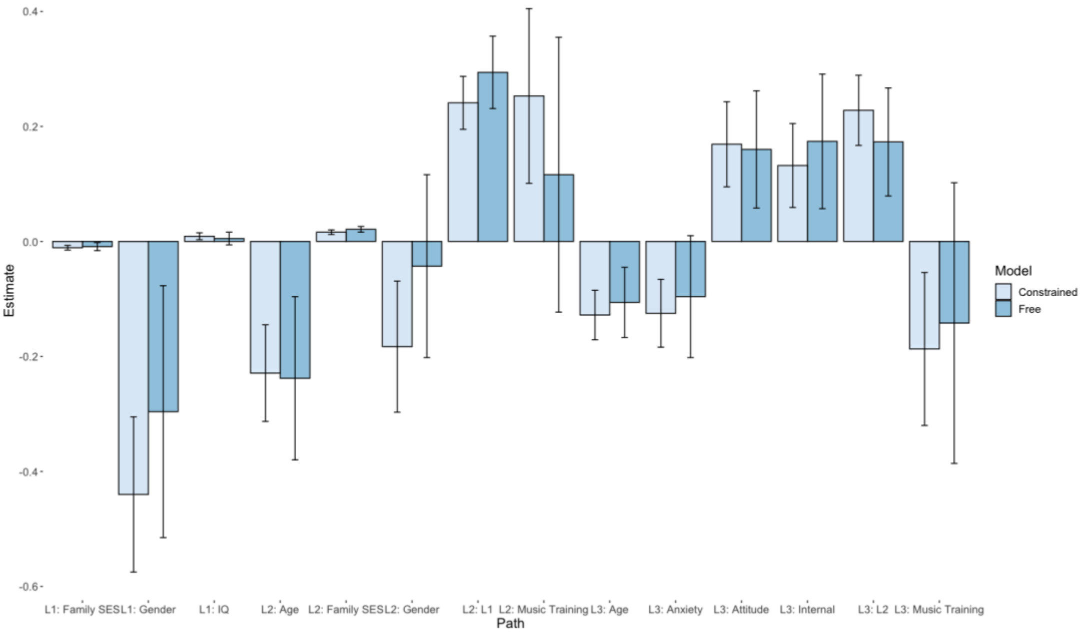

Spanish Learners

Supplementary Figure 5b: Contrasts of free" and "constrained" LCH models with L3 languages as the group comparison factor. a) German learners from the German vs. Spanish groups. b) Spanish learners from German vs. Spanish groups. Each comparison included a "free" model, in which all parameters are allowed to differ between the groups, and a "constrained" model, in which each path, regardless of its group, is calculated from the pooled data across groups. Since the two models did not significantly differ and the "constrained" model fitted the data well, we assume that there is no variation in the path coefficients depending on the L3 languages (refer to the Main Text for further description of the data). Error bars indicate standard errors.

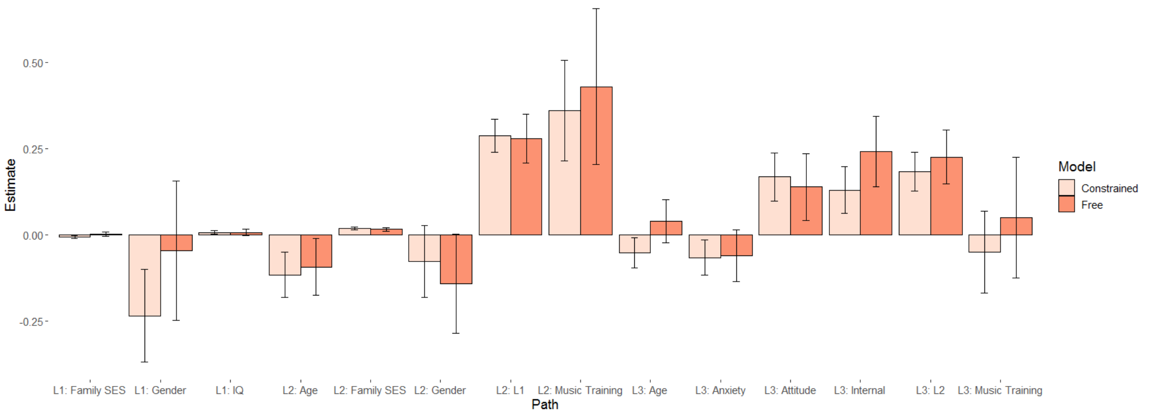

French Learners

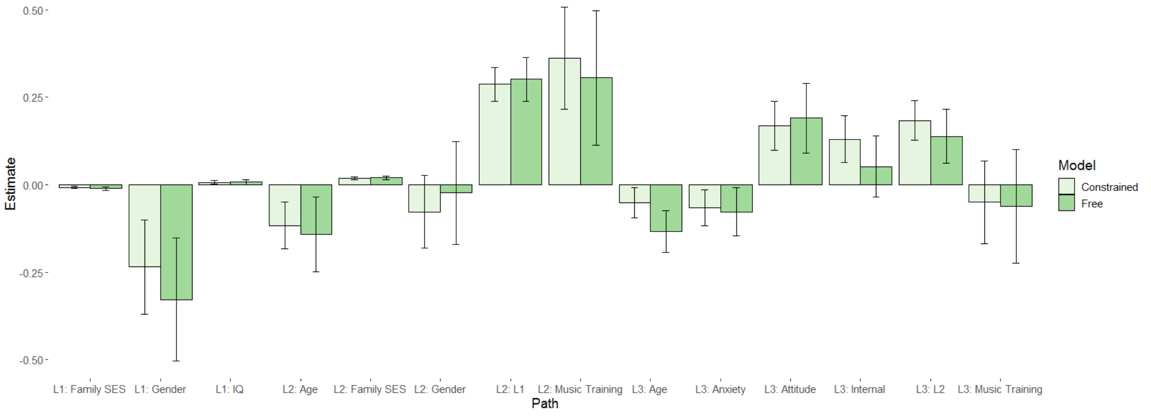

Spanish Learners

Supplementary Figure 5c: Contrasts of “free” and “constrained” LCH models with L3 languages as the group comparison factor. As there were no significant differences in all group comparisons, only one example of each group of learners is included: a) Spanish learners from French vs. Spanish groups. b) French learners from French vs. Spanish groups. Each comparison included a “free” model, in which all parameters are allowed to differ between the groups, and a “constrained” model, in which each path, regardless of its group, is calculated from the pooled data across groups. Since the two models did not significantly differ and the “constrained” model fitted the data well, we assume that there is no variation in the path coefficients depending on the L3 languages (refer to the Main Text for further description of the data). Error bars indicate standard errors.

Supplementary Table 1 Automatically extracted variables of spoken narratives using CLAN (adapted from the CLAN manual, see MacWhinney, 2016b; MacWhinney, 2000). In total, we obtained 15 measures from the raw data, which were then reduced to two components using the principal component analysis approach.

Variables		Definition
**Length and complexity** (7 variables)	Total utterances	The number of utterances that are used in computing mean length of utterances (MLU) plus unintelligible utterances. Non-word utterances are excluded.
	MLU utterances	The number of utterances that are used in computing mean length of utterances after excluding unintelligible utterances and non-word utterances.
	MLU words	MLU in words after excluding words in unintelligible utterances (marked as xxx, yyy, or www).
	MLU Morpheme	MLU in morpheme after excluding words in unintelligible utterances (marked as xxx, yyy, or www).
	Total Words	The number of words used for analysis for MOR.
	Density	Measure of propositional idea density based on Turner & Green (1977).
	Verbs per Utterances	The average number of verbs per utterance excluding modals.
**Lexical Diversity** (3 variables)	Types	Total word types excluding repetitions and revisions.
	Tokens	Total word tokens excluding repetitions and revision.
	MATTR	As the length of text affects the type/token ratio (longer samples tend to produce lower ratio values), we calculated the Moving-Window Type/Token Ratio (MATTR) as a measure of lexical diversity (Covington & McFall, 2010). We used 30 words as the fixed size window of the text.
**Accuracy** (2 variables)	Word Errors	The number of words that are coded as errors. Percentage was calculated for data analysis by dividing the number of word errors.
	Utterance Errors	The number of utterances coded as errors. Percentage was calculated for data analysis by dividing the number of word errors from the MLU utterances.
**Fluency** (3 variables)	Words per Minute	The number of words per minute.
	Retracing/Duration	The number of retracing including self-corrections or changes divided by the total duration.
	Repetition/Duration	The number of repetitions divided by the total duration.

Supplementary Table 2 Descriptive statistics of participants' third language proficiency measures.

Variables	French learners			German learners			Spanish learners		
	Mean (SD)	Range	Ns	Mean (SD)	Range	Ns	Mean (SD)	Range	Ns
L3 Pronunciation	0.00 (0.99)	-3.03 – 2.44	179	0.00 (0.99)	-2.51 – 2.58	176	0.00 (0.99)	-2.43 – 2.62	271
L3 Exam	0.11 (0.90)	-4.16 – 1.97	187	0.08 (0.92)	-2.76 – 2.80	176	0.10 (0.92))	-2.85 – 2.53	273
L3 Narrative Length	0.00 (0.99)	-2.26 – 4.40	182	0.00 (1.07)	-2.51 – 3.62	172	0.01 (0.95)	-2.27 – 4.16	257
L3 Narrative Content	0.00 (1.03)	-3.27 – 3.10	182	0.00 (0.85)	-2.66 – 3.06	172	0.00 (1.07)	- 2.65 – 3.31	257
L3 Language Access	0.00 (0.99)	-1.42 – 8.46	185	0.00 (0.99)	-1.93 – 3.00	171	0.00 (0.99)	-2.12 – 5.69	259

Supplementary Table 3 Pairwise Spearman's rank correlation coefficients for proficiency variables of L1, L2, and L3. Associated statistical significance was two-sided and adjusted for multiple tests using the false discovery rate (FDR) method. *$p < 0.05$, **$p < 0.01$, ***$p < 0.001$

		1	2	3	4	5	6	7	8
**1.**	**L3 Global**	1							
**2.**	**L3 Narrative Length**	0.50***	1						
**3.**	**L3 Narrative Content**	0.31***	0.00	1					
**4.**	**L3 Pronunciation Ratings**	0.61***	0.12**	0.19***	1				
**5.**	**L3 Language Access**	0.58***	0.21***	-0.01	0.16***	1			
**6.**	**L3 Classroom Exam**	0.64***	0.13**	0.11*	0.20***	0.26***	1		
**7.**	**L1 (Chinese) HKDSE grades**	0.05	0.08*	-0.06	-0.02	0.02	0.09	1	
**8.**	**L2 (English) HKDSE grades**	0.28***	0.09*	0.07	0.17***	0.16*	0.28***	0.26***	1
