# Supplementary material for: Language and nonlanguage factors in foreign language learning: evidence for the learning condition hypothesis
Source: NPJ Sci Learn. 2021 Sep 15;6:28. doi: 10.1038/s41539-021-00104-9 (PMC8443555; doi:10.1038/s41539-021-00104-9)
Supplement: Supplementary file 2 — Reporting Summary [file 41539_2021_104_MOESM2_ESM.pdf]

nature research

Corresponding author(s): Patrick CM Wong

Last updated by author(s): 31/7/21

Reporting Summary

Nature Research wishes to improve the reproducibility of the work that we publish. This form provides structure for consistency and transparency in reporting. For further information on Nature Research policies, see our Editorial Policies and the Editorial Policy Checklist.

Statistics

For all statistical analyses, confirm that the following items are present in the figure legend, table legend, main text, or Methods section.

n/a	Confirmed	
☐	☒	The exact sample size (*n*) for each experimental group/condition, given as a discrete number and unit of measurement
☐	☒	A statement on whether measurements were taken from distinct samples or whether the same sample was measured repeatedly
☐	☒	The statistical test(s) used AND whether they are one- or two-sided<br>*Only common tests should be described solely by name; describe more complex techniques in the Methods section.*
☐	☒	A description of all covariates tested
☐	☒	A description of any assumptions or corrections, such as tests of normality and adjustment for multiple comparisons
☐	☒	A full description of the statistical parameters including central tendency (e.g. means) or other basic estimates (e.g. regression coefficient) AND variation (e.g. standard deviation) or associated estimates of uncertainty (e.g. confidence intervals)
☐	☒	For null hypothesis testing, the test statistic (e.g. *F*, *t*, *r*) with confidence intervals, effect sizes, degrees of freedom and *P* value noted<br>*Give P values as exact values whenever suitable.*
☒	☐	For Bayesian analysis, information on the choice of priors and Markov chain Monte Carlo settings
☐	☒	For hierarchical and complex designs, identification of the appropriate level for tests and full reporting of outcomes
☐	☒	Estimates of effect sizes (e.g. Cohen's *d*, Pearson's *r*), indicating how they were calculated

Our web collection on statistics for biologists contains articles on many of the points above.

Software and code

Policy information about availability of computer code

Data collection	E-prime, Qualtrics, & Google Forms
Data analysis	R and Rstudio, including the e1071, caret, lavaan, ggplot2 packages.

For manuscripts utilizing custom algorithms or software that are central to the research but not yet described in published literature, software must be made available to editors and reviewers. We strongly encourage code deposition in a community repository (e.g. GitHub). See the Nature Research guidelines for submitting code & software for further information.

Data

Policy information about availability of data

All manuscripts must include a data availability statement. This statement should provide the following information, where applicable:

- Accession codes, unique identifiers, or web links for publicly available datasets
- A list of figures that have associated raw data
- A description of any restrictions on data availability

All data needed to evaluate the conclusions in the paper are present in the paper and/or the Supplementary Materials. The numeric data and analysis scripts of this study will be available at Open Science Framework link: https://osf.io/f5wt8/

Field-specific reporting

Please select the one below that is the best fit for your research. If you are not sure, read the appropriate sections before making your selection.

☐ Life sciences ☒ Behavioural & social sciences ☐ Ecological, evolutionary & environmental sciences

For a reference copy of the document with all sections, see nature.com/documents/nr-reporting-summary-flat.pdf

Behavioural & social sciences study design

All studies must disclose on these points even when the disclosure is negative.

Study description	Qualitative cross-sectional
Research sample	Participants were 636 undergraduate students between 18-25 years old studying at the Chinese University of Hong Kong (CUHK). All participants were sequential bilinguals who learned Cantonese as their native language and learned English as L2 in the formal education system since around 3 years old. All participants were university students who learned L3 for college credits at the time of participation in this research.
Sampling strategy	Participants were recruited using convenience sampling method. Participants were invited to join the study through mass emails and advertisements in their language classes after obtaining permission from the language teachers. Based on the minimum significant correlation value we obtained (that between L1 and L2, r = 0.26) and for a family-wise alpha of 0.05 (Bonferroni-corrected p-value of 0.017 for three tests performed), a minimum of 150 participants were required. For each language, we have data from at least 167 participants for the key measures of L1, L2 and L3 proficiency. Our study is therefore sufficiently powered.
Data collection	We have three types of data. 1. L1 and L2 proficiency measures were collected using participants' admission record. 2. L3 classroom exam scores were provided by the department. 3. We obtained demographics information and L3 outcomes measures in the laboratory. Participants filled in questionnaires themselves. Nonverbal IQ test was administered by trained research assistants. Narrative sample in L3 and body part naming task were administered using Eprime.
Timing	04/Jan/2014 -18/Dec/2017 (almost 4 years)
Data exclusions	We excluded participants who did not have a HKDSE score. These participants may be enrolled in CUHK via IB exam, for example.
Non-participation	NA
Randomization	NA

Reporting for specific materials, systems and methods

We require information from authors about some types of materials, experimental systems and methods used in many studies. Here, indicate whether each material, system or method listed is relevant to your study. If you are not sure if a list item applies to your research, read the appropriate section before selecting a response.

Materials & experimental systems

n/a	Involved in the study	
☒	☐	Antibodies
☒	☐	Eukaryotic cell lines
☒	☐	Palaeontology and archaeology
☒	☐	Animals and other organisms
☐	☒	Human research participants
☒	☐	Clinical data
☒	☐	Dual use research of concern

Methods

n/a	Involved in the study	
☒	☐	ChIP-seq
☒	☐	Flow cytometry
☒	☐	MRI-based neuroimaging

Human research participants

Policy information about studies involving human research participants

Population characteristics	See above. None of these participants have any neurological disorders or abnormal hearing. All of them had nonverbal IQ within normal limits (≥ 85), as assessed by the Test of Nonverbal Intelligence, Fourth Edition (TONI-4). 74% of participants are female and 26% are male
Recruitment	See above.

Ethics oversight	The research protocol was approved by the Joint Chinese University of Hong Kong – New Territories East Cluster Clinical Research Ethics Committee.

Note that full information on the approval of the study protocol must also be provided in the manuscript.
